# WDR72 Promotes Neuroblastoma Stemness and Progression by Sequestering TRIM31‐Mediated Degradation of CBX8

**DOI:** 10.1002/advs.76602

**Published:** 2026-07-30

**Authors:** Huijuan Zeng, Zijie Ye, Manna Zheng, Jing Pan, Tianbao Tan, Jiliang Yang, Jiahao Li, Zhang Zhao, Tianzhu Long, Liyu Zhang, Gautam Sethi, Tianyou Yang, Chao Hu, Yan Zou

**Affiliations:** ^1^ Department of Pediatric Surgery, Guangzhou Institute of Pediatrics, Guangzhou Women and Children's Medical Center, Guangdong Provincial Clinical Research Center For Child Health Guangzhou Medical University Guangzhou Guangdong China; ^2^ Thyroid and Breast Surgery Center, Guangzhou Women and Children's Medical Center, Guangdong Provincial Clinical Research Center For Child Health Guangzhou Medical University Guangzhou Guangdong China; ^3^ Department of Pharmacology Yong Loo Lin School of Medicine National University of Singapore Singapore Singapore; ^4^ NUS Centre for Cancer Research Yong Loo Lin School of Medicine National University of Singapore Singapore Singapore

**Keywords:** CBX8, competitive modification of ubiquitination, neuroblastoma, stemness, WDR72

## Abstract

Cancer stem cells are pivotal in cancer progression, yet the mechanisms underlying the stemness properties of neuroblastoma cells remain largely unclear. By analyzing data from the TARGET‐NBL and GEO databases, WDR72 is identified as a cancer stem cell marker that significantly correlates with high‐risk of neuroblastoma and poor prognosis in neuroblastoma patients. It is found that knocking down WDR72 in neuroblastoma cells suppresses cell proliferation and migration, while inducing apoptosis. An increased expression of WDR72 is detected in neuroblastoma cells with enhanced stemness properties, while silencing WDR72 reduces the stemness of these cells. Mechanistically, we find that the upregulation of WDR72 is mediated by m6A modifications in its 3’ UTR induced by the METTL14‐IGF2BP1 axis. Further investigation into the downstream regulatory mechanisms of WDR72 demonstrates that WDR72 interacts with TRIM31, blocking the ubiquitination and subsequent degradation of the CBX8 protein. This interaction stabilizes CBX8, contributing to the maintenance of neuroblastoma cell stemness. This study reveals that the upregulation of WDR72 via the METTL14‐IGF2BP1 axis enhances the stemness properties of neuroblastoma cells by stabilizing CBX8 through interaction with TRIM31. These findings provide new insights into the molecular mechanisms driving neuroblastoma malignancy and offer potential therapeutic targets for patients with neuroblastoma.

## Graphical abstract

This study unveiled that METTL14 mediates m6A modification of WDR72 mRNA to stabilize and enhance WDR72 expression, which disrupts TRIM31‐mediated ubiquitination of CBX8 protein and retards its degradation, finally the elevated CBX8 contributes to tumor stemness.

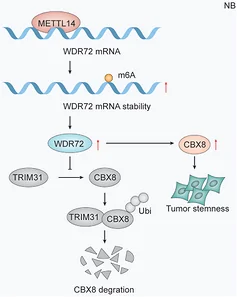

## 1 Introduction

Neuroblastoma is the most common extracranial solid tumor in children and a leading cause of cancer‐related mortality in pediatric patients [1]. This malignancy arises from neural crest cells, and develops into a heterogenous pediatric tumor of the sympathetic nervous system [2]. Neuroblastoma primarily affects infants and young children, displaying a wide spectrum of clinical behaviors, ranging from spontaneous regression to aggressive metastatic disease. Prognosis is highly variable in neuroblastoma, with high‐risk patients associated with poorer outcomes compared to the low/intermediate‐risk ones [3].

The heterogeneous nature of neuroblastoma has made it a major focus of research, particularly regarding cancer stem cells (CSCs) or tumor‐initiating cells. CSCs share properties with normal stem cells, including the ability to self‐renew and differentiate into multiple tumor cell types. These cells are considered key drivers of tumor initiation, progression, metastasis, and resistance to conventional therapies [4]. In neuroblastoma, the presence of CSCs complicates treatment, as they can survive chemotherapy and contribute to tumor relapse and metastasis. Understanding the characteristics and behavior of CSCs in neuroblastoma is crucial for developing targeted therapies that effectively eradicate these cells and improve patient outcomes.

Research efforts increasingly focus on identifying specific markers and signaling pathways associated with CSCs in neuroblastoma. These markers provide insights into the mechanisms underlying CSC maintenance and offer potential therapeutic targets [5, 6]. Emerging strategies include the use of small molecules, monoclonal antibodies, and gene therapies to selectively eliminate or inhibit CSCs. Targeting these cells aims to develop more effective and less toxic treatments for neuroblastoma patients.

One promising research area involves WD repeat domain 72 (WDR72), a member of the WD40‐repeat domain superfamily [7]. Proteins in this family are implicated in various cellular processes, including cell cycle regulation, signal transduction, and transcription. While WDR72 has primarily been associated with amelogenesis imperfecta, a genetic disorder affecting tooth enamel development [8, 9], recent findings suggest an oncogenic role in lung cancer, where it promotes stemness and tumor progression [10, 11]. Besides, a report also revealed WDR72 as a CSC‐related gene in gastric cancer [12]. However, the role of WDR72 in neuroblastoma remains largely unexplored.

In this study, we aimed to investigate the role of WDR72 in neuroblastoma by analyzing data from the TARGET‐N BL and GEO databases. Our preliminary analysis identified WDR72 as a potential marker for CSCs in neuroblastoma, with its expression correlating with high‐risk neuroblastoma and poor prognosis. This suggests that WDR72 may play a significant role in the malignant progression of neuroblastoma, potentially through its influence on CSC properties. To our knowledge, the specific role of WDR72 in neuroblastoma has not been previously reported.

The primary objective of this study is to elucidate the function of WDR72 in regulating CSC‐induced malignant progression in neuroblastoma cells. We hypothesize that WDR72 contributes to the maintenance and proliferation of CSCs, thereby promoting tumor aggressiveness and treatment resistance. Additionally, we aim to explore the upstream and downstream regulatory mechanisms associated with WDR72, including its potential interactions with other proteins and signaling pathways involved in neuroblastoma pathogenesis. By gaining a deeper understanding of WDR72's involvement in neuroblastoma, we hope to contribute to the understanding of neuroblastoma pathogenesis and identify novel therapeutic targets for this aggressive pediatric malignancy. Our findings could pave the way for the development of targeted therapies that specifically address the CSC population in neuroblastoma, ultimately improving treatment outcomes and survival rates for affected children.

## 2 Methods

### 2.1 Clinical Samples

Tissue samples from 17 cases of metastatic neuroblastoma and 9 cases of non‐metastatic neuroblastoma were collected by Guangzhou Women and Children's Medical Center. These samples were used to analyze the expression of focused genes via qPCR and western blot. The clinical, prognostic, and pathological information of the patients was included. The sponsors fully understood and agreed to the research. The procedures of this study were approved by the Research Ethics Committee of Guangzhou Women and Children's Medical Center (No.[2023]064B00) and conducted in accordance with the Code of Ethics of the World Medical Association. The experimental methods comply with the Helsinki Declaration.

### 2.2 Tissue Microarray

The tissue microarray analyses were accomplished by bioaitech company, with the microarray numbered N264001. The microarray included 22 cases of neuroblastoma tissue and 4 cases of normal adrenal tissue from healthy donors, and 8 of 22 neuroblastoma cases were with lymph node metastasis or distant metastasis.

### 2.3 Bioinformatics Analyses

TARGET‐NBL database was applied to obtained genes with high risk or poor prognosis of neuroblastoma patients. GSE90789 was used to analyze differential genes in spheres of tumors from relapsed and newly‐diagnosed neuroblastoma patients. Since CSCs are able to isolated as tumor sphere, we set those genes upregulated in relapsed spheres as CSCs markers. Genes related to high risk of neuroblastoma patients was selected via Log2FC>1.5 and adjP<0.05. Genes correlated with poor prognosis of neuroblastoma patients were screened through HR>0.3, P<0.05, and PH>0.1. CSCs markers were screened via log2FC>1.5. The genes associated with high risk or poor prognosis of neuroblastoma patients obtained from TARGET‐NBL, and CSCs markers acquired from GSE90789, were all listed in Supplementary table 1.

### 2.4 Animals

All experiments were performed in accordance with the Guide for the Care and Use of Laboratory Animals, and authorized by Guangzhou Women and Children's Medical Center (No. G20230376). Six to eight‐week‐old NOD/SCID/IL‐2Ry‐null (NSG) mice were purchased from Guangdong Medical Laboratory Animal Center and maintained under specific pathogen‐free conditions. Specifically, the air humidity was maintained between 40% and 70%. The temperature was controlled at approximately 22°C, with a permissible fluctuation of ±2°C. Additionally, we employed a light/dark cycle of 12 h each. Regarding their diet, the mice were provided with standard sterile food and water ad libitum. Subcutaneous xenograft models were established by injecting 2×10^6^/100 µl SK‐N‐SH cells in PBS into the left axilla of each mouse (n = 5 each group). The tumors were recorded every three days from Day 7 to Day 28, and the tissues were acquired and weighted after mice sacrifice. The in vivo metastasis model was set by injecting 2×10^7^ IMR‐32 cells transfected with shNC or shWDR72 into the NSG mice (n = 4 each group) via the tail vein. Four weeks later, mice were sacrificed and the lungs were obtained to analyze the metastatic nodules. The orthotopic neuroblastoma animal model was established based on previous experimental methods [13]. Five‐week‐old female SCID mice were used for this model. Different treated IMR‐32 cells were resuspended in HEPES buffer to a concentration of 1.25 × 10^7^ cells mL^−1^. A total of 20 µl of the cell suspension was injected into the adrenal gland of the left kidney of each mouse (*n* = 4 each group). The mice were monitored twice weekly for their health status after the injection. Tissue samples were collected in the third week of modeling for subsequent experiments. This study was conducted with ethical approval and review.

### 2.5 Cell Culture and Treatment

Two neuroblastoma cell lines (SK‐N‐SH and IMR‐32) and 293T cells were obtained from the American Type Culture Collection (ATCC, Manassas, VA, USA). Routine short tandem repeat (STR) testing was performed to confirm the authenticity of the cell lines and exclude contamination. Cells were cultured in Dulbecco's Modified Eagle Medium (DMEM) and maintained at 37°C in a humidified atmosphere with 5% CO_2_. Cells were treated with 20 µM MG132 for 4 h, 50 µg mL^−1^ cycloheximide (CHX) for 0, 6, and 12 h, and 100 nM bafilomycin A1 (BafA1) for 1 h.

### 2.6 Transfections

Short hairpin RNAs (shRNAs) targeting WDR72 (sh‐WDR72#1 and sh‐WDR72#2) and METTL14 (sh‐METTL14#1 and sh‐METTL14#2) were synthesized by GenePharma (Jiangsu, China). The full sequences of WDR72, METTL14, and TRIM31 were synthesized and cloned into pcDNA 3.1 vectors to generate corresponding overexpression constructs. For transient transfections, siRNAs or plasmids were mixed with Lipofectamine 3000 (Invitrogen, Carlsbad, CA, USA) in OptiMEM (Gibco) to form complexes, and then transfected into cells for 48 h. Cells were subsequently harvested for further experiments.

### 2.7 Quantitative Reverse Transcription Real‐Time PCR (RT‐qPCR)

Total RNA was extracted using TRIzol (TransGen Biotech, China) according to the manufacturer's protocol. Complementary DNA (cDNA) synthesis was performed using M‐MLV reverse transcriptase (Vazyme, Nanjing, China). RT‐qPCR was conducted with SYBR Green master mix (Vazyme) on an ABI Prism 7500 Sequence Detector (Applied Biosystems, USA). mRNA levels were normalized to the internal control gene GAPDH, and relative mRNA expression levels were calculated using the 2^−ΔΔCt^ method [14].

### 2.8 Cell Counting Kit‐8 (CCK‐8) Assay

For the CCK‐8 assay, cells were seeded in a 96‐well plate at a density of 2 × 10^3^ cells per well and incubated for 24 h. CCK‐8 solution (10 µL; Beyotime, China) was added to each well at 24, 48, and 96 h, followed by incubation for 2 h at 37°C. Absorbance at 450 nm was measured using a microplate reader.

### 2.9 Colony Formation Assay

Cells were seeded in 6‐well plates at a density of 500 cells per well to assess proliferation. After 14 days, colonies were washed with phosphate‐buffered saline (PBS), fixed with 70% ethanol, and stained with 0.5% crystal violet. Colonies were counted and recorded.

### 2.10 Transwell Assay

Cells transfected with the indicated plasmids were starved in serum‐free medium. A cell suspension of 0.2 mL DMEM was added to the upper chamber of a Transwell insert (8‐µm pore size, Corning, New York, USA) pre‐coated with 50 µl Matrigel (BD Biosciences, San Jose, CA, USA) for the invasion assay, or uncoated for the migration assay. DMEM containing 10% fetal bovine serum (FBS) was added to the lower chamber. After 48 h of incubation, non‐migrated cells were removed from the upper chamber using a cotton swab. Cells that had migrated or invaded to the lower side of the upper chamber were fixed with methanol, stained with 2% crystal violet for 10 min, and counted in five random fields under a microscope.

### 2.11 Flow Cytometry Assay

Cells were treated as indicated, incubated for 48 h, and harvested for flow cytometry. Apoptosis was assessed using an Annexin V‐FITC/PI apoptosis detection kit (Multi Sciences, Hangzhou, China) following the manufacturer's protocol, and analyzed with a flow cytometer (BD Biosciences).

### 2.12 Sphere Formation Assay

Harvested cells were resuspended as single cells in serum‐free medium and seeded into 96‐well plates at a density of 200 cells per well, with each well containing 200 µl of serum‐free medium. Medium was changed every 2 days. Sphere formation was observed and recorded using an optical microscope (Leica, Wetzlar, Germany).

### 2.13 Western Blot

Cells were lysed using RIPA buffer (CWBio, Beijing, China) containing protease inhibitors. Lysates were mixed with loading buffer and denatured at 100°C for 10 min. Proteins were separated by 10% SDS‐PAGE and transferred to polyvinylidene difluoride (PVDF) membranes (Millipore, Billerica, MA, USA). Membranes were blocked and incubated overnight at 4°C with primary antibodies: anti‐WDR72, anti‐ALDH1, anti‐Nanog, anti‐CBX8, anti‐TRIM31 (all 1:1000, Abcam, USA), and anti‐GAPDH (1:1000, Abcam). Secondary antibody incubation followed (1:2000, Abcam), and bands were visualized using ECL substrate (Millipore).

### 2.14 RIP Assay

RIP assay was performed using Protein A/G Agarose Resin (YEASEN, China). Cell lysates were extracted with NP‐40 lysis buffer (Beyotime, China), and 100 µL lysate was incubated with NP‐40 buffer containing Protein A/G beads conjugated to anti‐m6A, anti‐METTL3, anti‐METTL14, or anti‐METTL16 antibodies at 4°C overnight. Protein‐RNA complexes were isolated, digested with protease K (YEASEN), and RNA was extracted for RT‐qPCR to identify the enrichment of WDR72 3'UTR.

### 2.15 Coimmunoprecipitation (Co‐IP) Assay and Mass Spectrometry

Cell lysates were incubated with primary antibodies (anti‐WDR72, anti‐TRIM31, and anti‐CBX8) or rabbit IgG (negative control) at 4°C overnight. Protein A/G agarose (20 µl; Bioworld Technology, St. Louis Park, MN, USA) was added and incubated at 4°C for 2 h. After washing, Protein‐antibody complexes were collected by centrifugation and subjected to Western blot or mass spectrometry analysis as previously described [15]. The data reported in this paper have been deposited in the OMIX, China National Center for Bioinformation / Beijing Institute of Genomics, Chinese Academy of Sciences (https://ngdc.cncb.ac.cn/omix: accession numbers OMIX010833, OMIX010974) [16, 17].

### 2.16 Immunofluorescence Assay

Cells were processed, fixed, and incubated with specific primary antibodies including anti‐CBX8 (PA5‐83193, 1 µg mL^−1^, Invitrogen), anti‐TRIM31 (12543‐1‐AP, 1:200 dilution, Proteintech), anti‐Ub‐K48 (ab140601, 1:500 dilution, Abcam), anti‐Ub‐K63 (14‐6077‐82, 1:300 dilution, Invitrogen), anti‐WDR72 (ab222307, 4 µg mL^−1^, Abcam), followed by washing and incubation with fluorescent‐labeled secondary antibodies. Samples were washed, stained, and observed under a fluorescence microscope.

### 2.17 Immunohistochemistry (IHC)

The metastatic and non‐metastatic neuroblastoma tissues subjected to fixation via 4% paraformaldehyde were cut into slices of 4 µm‐thickness. Following being dewaxed by xylene and hydrated by gradient ethanol, the tissue slides were boiled for 20 min and cooled down. After that, the slides were treated with primary antibodies including anti‐TRIM31 (12543‐1‐AP, 1:200 dilution, Proteintech) and anti‐CBX8 (PA5‐83193, 1:50 dilution, Invitrogen) for 1 h at room temperature, followed by incubation with HRP‐coupled secondary antibody at room temperature for another 1 h. The staining of TRIM31 or CBX8 was visualized via 3,3‐N‐diaminobenzidine (DAB), and hematoxylin was used to counterstain the nuclei. Following dehydrated, cleared, and sealed, the samples were observed under an inverted microscope. The percentage of stained area in total area were calculated as staining positivity.

### 2.18 M6A Dot Blot Assay

The m6A dot blot assay was conducted as previously described [18]. In brief, after being dissolved in 3 times volume of RNA incubation buffer and denatured, RNA samples were divided into subgroups of 400, 200, and 100 ng, and then mixed with ice‐cold 20*SSC buffer (Sigma–Aldrich, Germany). After that, the samples in different subgroups were all loaded to an Amersham Hybond‐N+ membrane (GE Healthcare, USA) installed in a Bio‐Dot Apparatus (Bio‐Rad, USA). Then, the membrane was UV crosslinked, washed, stained with 0.02% Methylene blue (Sangon Biotech, China), scanned, and blocked with BSA, followed by incubation with specific m6A antibody (68055‐1‐Ig, 1:2000 dilution, Proteintech) overnight at 4°C. After further incubation with HRP‐conjugated secondary antibody for 1 h at room temperature, the dot blots were visualized via an imaging system (Bio‐Rad, USA). The integrated density of each dot was counted by ImageJ, and relative m6A level was calculated as the ratio of integrated density of experimental dots to that of control dots.

### 2.19 Ubiquitination Assay

To detect polyubiquitinated CBX8, whole cell lysates were prepared with RIPA buffer containing protease inhibitors. Lysates were subjected to immunoprecipitation of endogenous CBX8, and ubiquitination levels were analyzed by Western blot using an anti‐Ub antibody.

### 2.20 GST Pull Down

To validate protein binding in vitro, TRIM31 was tagged with a His tag and CBX8 was tagged with a GST tag, and both were expressed in engineered 293T cells. After lysing the cells, the supernatant was collected by centrifugation, and the purified GST fusion protein (bait protein) was obtained using glutathione agarose beads. The bait protein‐GSH magnetic bead complex was then incubated with the lysate of His‐TRIM31‐overexpressing cells for 3–4 h. Subsequently, the bait‐captured protein complex was eluted, and the eluted products were analyzed by Western blot. The experiment was conducted using the GST pull‐down kit (Agarose) (Elabscience, Wuhan, China, EA‐IP‐K008), following the protocol provided in the kit instructions.

### 2.21 Dual‐luciferase Reporter Assay

After constructing the 3’ UTR region of WDR72 into the psicheck2 vector, the psicheck2‐WDR72 vector was co‐transfected with METTL14 or IGF2BP1 vectors into adherent cells using Lipofectamine 2000 (Invitrogen). After 48 h of transfection, relative luciferase activity was measured using a dual‐luciferase reporter assay system (Meilunbio, Dalian, China).

### 2.22 Statistical Analysis

Experiments were conducted in triplicate, and results are presented as means ± standard deviation (SD). Statistical analyses were performed using SPSS 22.0 software (IBM, USA). Student's t‐test was used to compare two groups, while one‐way ANOVA or Two‐way repeated measures ANOVA was used for multiple groups. A p‐value <0.05 was considered statistically significant. We declared that no other scripts and software were used other than those mentioned in the methods section.

## 3 Results

### 3.1 WDR72 Expression Correlates with Neuroblastoma Malignancy

To search factors significant in neuroblastoma, we began our study by analyzing data from the TARGET‐NBL and GSE90789 databases. Such analyses highlighted four potential CSC markers (including KIRREL2, NCAN, EPPK1 and WDR72) which were simultaneously associated with high‐risk and poor prognosis in neuroblastoma patients (Figure 1A). The survival curve of neuroblastoma patients with high or low expressions of above four factors were shown in Additional file 1: Figure S1. Next, we collected tissue samples from 9 non‐metastatic and 17 metastatic neuroblastoma cases. Our analysis revealed that only WDR72 among above four genes showed a significant upregulation in metastatic tissues compared to non‐metastatic samples (Figure 1B). This observation was further supported by Western blot analysis, which demonstrated elevated WDR72 expression in randomly selected metastatic tissues (6 non‐metastatic and 6 metastatic cases) (Figure 1C). Additionally, tissue microarray analysis confirmed the marked upregulation of WDR72 in metastatic neuroblastoma tissues (Figure 1D). To further elucidate the role of WDR72 in metastatic neuroblastoma, we evaluated the expression levels of stem cell markers ALDH1 and Nanog in these samples (Figure 1E). The analysis revealed significantly higher expression of both ALDH1 and Nanog in metastatic tissues, suggesting that WDR72 may enhance stemness and promote the maintenance of stem‐like characteristics in neuroblastoma cells.

**FIGURE 1:**
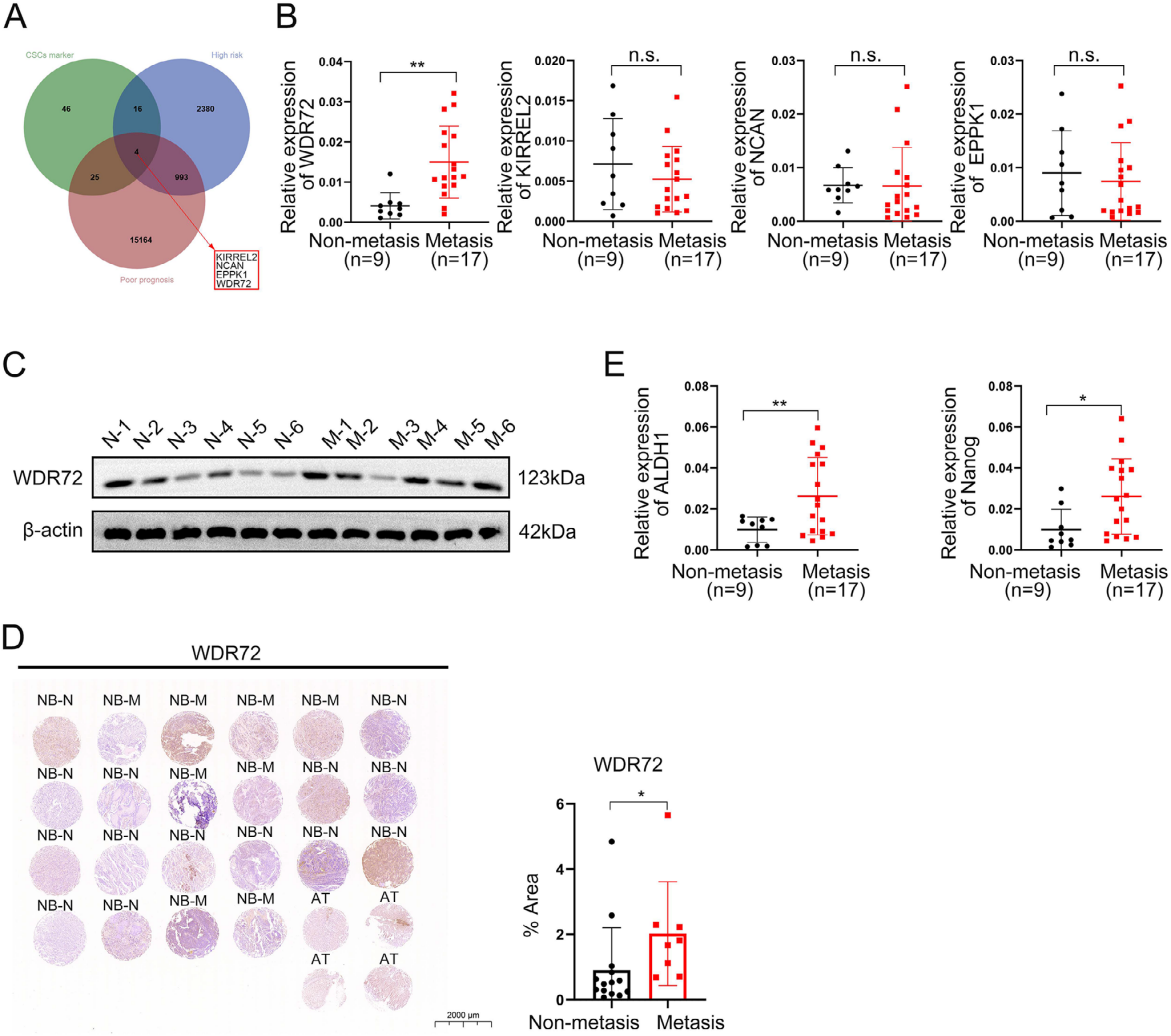
WDR72 Expression Correlates with neuroblastoma Malignancy. (A) Database analysis for screening differentially expressed genes to identify potential CSC markers. (B) RT‐qPCR detection of WDR72, KIRREL2, NCAN, and EPPK1 expression in neuroblastoma tissues. (C) Western blot analysis of WDR72 expression in randomly selected non‐metastatic (N) and metastatic (M) tissues (6 samples each). (D) Tissue microarray evaluation of WDR72 expression in neuroblastoma metastatic tissues, neuroblastoma non‐metastatic tissues, and adrenal gland tissues. (E) Expression levels of stem cell markers ALDH1 and Nanog in metastatic and non‐metastatic neuroblastoma tissues. Student's t‐test was used to analyze data of B, D and E. n.s.: not significant, ^*^ *p*<0.05, ^**^ *p*<0.01.

In summary, our findings demonstrate that WDR72 is abnormally overexpressed in metastatic neuroblastoma tissues, establishing a strong association between WDR72 and neuroblastoma malignancy.

### 3.2 WDR72 Promotes Proliferation, Invasion, Metastasis, and Stemness While Inhibiting Apoptosis in Neuroblastoma

To elucidate the functional role of WDR72 in neuroblastoma, we first performed gene silencing experiments in two neuroblastoma cell lines (Additional file 1: Figure S2A). Functional assays revealed that WDR72 silencing led to a marked reduction in cell viability and proliferation, as demonstrated by CCK‐8 (Additional file 1: Figure S2B) and colony formation assays (Additional file 1: Figure S2C). Furthermore, WDR72 knockdown significantly increased apoptosis rates (Additional file 1: Figure S2D) and impaired the migratory and invasive capacities of neuroblastoma cells, as shown by transwell assays (Additional file 1: Figure S2E,F).

In vivo, subcutaneous xenograft experiments in nude mice demonstrated that WDR72 knockdown significantly inhibited tumor growth (Figure 2A,B), while tail vein metastasis assays revealed a marked suppression of lung metastasis (Figure 2C,E), further supporting its role in promoting neuroblastoma malignancy. Additionally, we employed orthotopic neuroblastoma models to further validate our findings. WDR72 knockdown significantly suppressed tumor growth in the orthotopic setting (Figure 2F), further underscoring its critical role in neuroblastoma progression.

**FIGURE 2:**
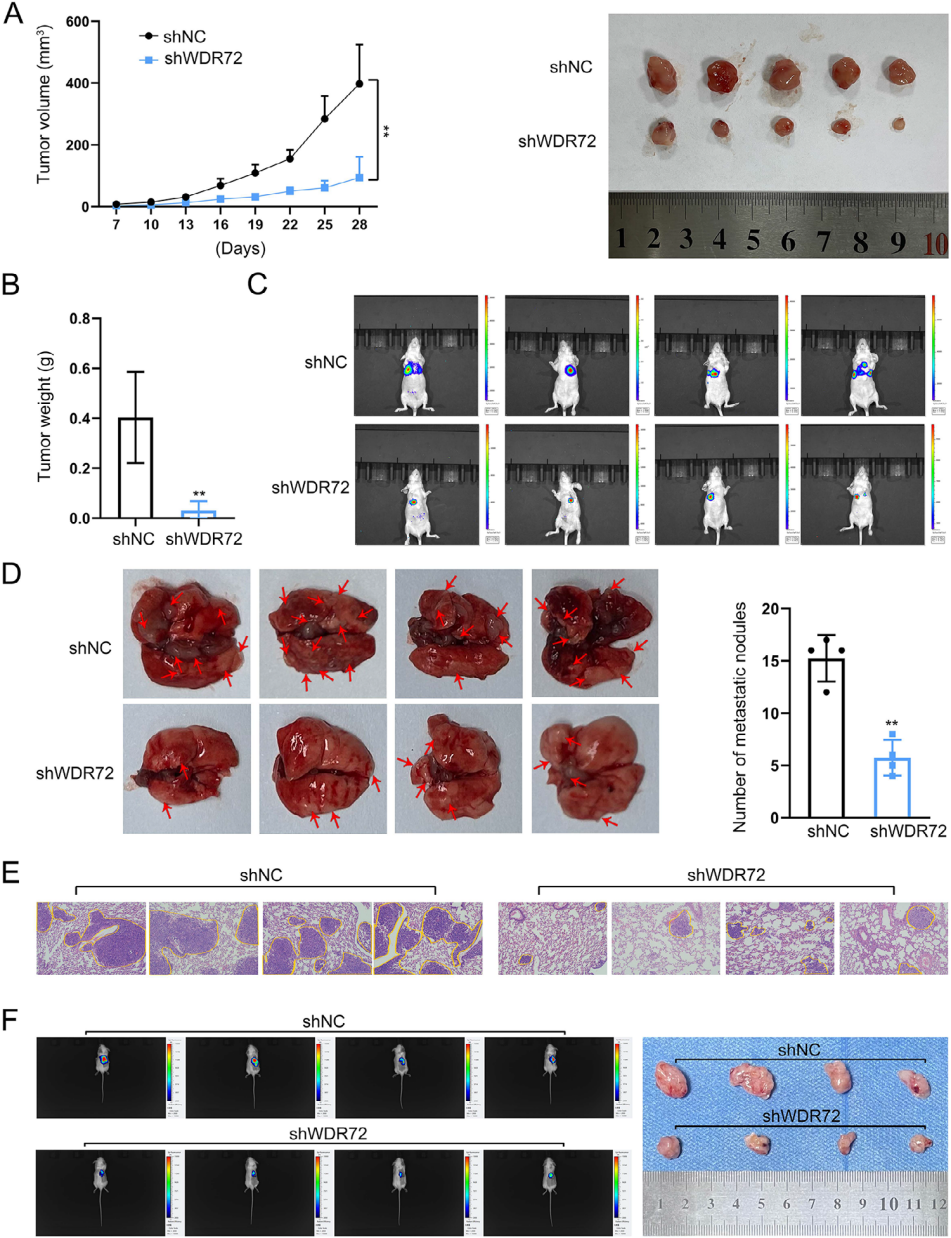
WDR72 Promotes neuroblastoma Growth and Metastasis In Vivo. (A) Interfere with WDR72, construct subcutaneous xenografts in nude mice, and analyze tumor growth across different groups. (B) Measure and compare tumor weight changes among different groups. (C) Construct a tail vein metastasis model and analyze fluorescence results to assess metastasis in various groups. (D) Evaluate and quantify lung metastatic nodules in different groups. (E) Perform HE staining to examine and analyze tumor lung metastasis in various groups. (F) Orthotopic neuroblastoma model demonstrating the suppression of tumor growth upon WDR72 knockdown. Two‐way repeated measures ANOVA was used to analyze data of A. Student's t‐test was used to analyze data of B and D. ^**^ *p*<0.01.

To further investigate the role of WDR72 in tumorigenicity—a hallmark of cancer stemness—we overexpressed WDR72 and conducted subcutaneous xenograft experiments in nude mice. Strikingly, the WDR72 overexpression group exhibited significantly enhanced tumorigenicity, as evidenced by accelerated tumor growth and increased tumor burden compared to controls (Additional file 1: Figure S3). These findings collectively highlight the crucial role of WDR72 in driving proliferation, invasion, metastasis, and tumorigenicity while inhibiting apoptosis in neuroblastoma.

### 3.3 WDR72 Enhances Neuroblastoma Cell Stemness

Given the established role of WDR72 as a CSC marker in neuroblastoma, we investigated its contribution to neuroblastoma malignancy by examining its regulatory effects on stemness. To this end, we compared adherent neuroblastoma cells with spheroid neuroblastoma cells, as spheroids are widely recognized to exhibit enhanced stem‐like properties. Considering that spheroid cells possess greater stemness potential and that higher‐passage spheroids display even stronger stemness characteristics, we aimed to elucidate the differential effects of WDR72 in adherent and spheroid neuroblastoma cells.

First, we established spheroid models of varying passages and analyzed WDR72 expression in these models (Figure 3A). RT‐qPCR analysis revealed a significant upregulation of WDR72 in neuroblastoma cell spheroids, with higher‐passage spheroids exhibiting progressively increased WDR72 expression. These findings suggest a strong correlation between WDR72 expression levels and the stemness potential of neuroblastoma cells.

**FIGURE 3:**
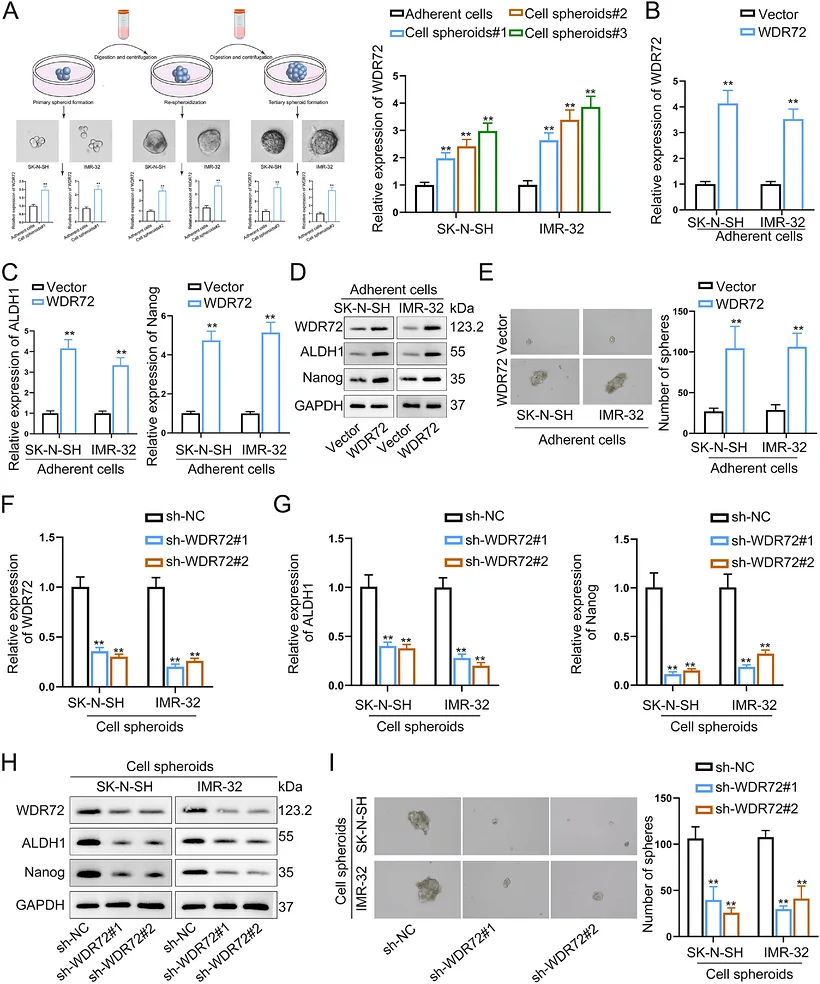
WDR72 Enhances neuroblastoma Cell Stemness. (A) Schematic diagram of spheroid models at various passages and RT‐qPCR analysis of WDR72 expression in these spheroids. (B) Overexpression of WDR72 in adherent cells, followed by RT‐qPCR to verify WDR72 overexpression efficiency. (C) RT‐qPCR detection of stemness markers ALDH1 and Nanog expression levels in adherent cells after WDR72 overexpression. (D) Western blot analysis of stemness markers ALDH1 and Nanog expression levels in adherent cells after WDR72 overexpression. (E) Sphere formation assay to evaluate the sphere‐forming ability of adherent cells after WDR72 overexpression. (F) Knockdown of WDR72 in cell spheroids, followed by RT‐qPCR to verify WDR72 knockdown efficiency. (G) RT‐qPCR detection of stemness markers ALDH1 and Nanog expression levels in sphere‐forming cells after WDR72 knockdown. (H) Western blot analysis of stemness markers ALDH1 and Nanog expression levels in cell spheroids after WDR72 knockdown. (I) Sphere formation assay to evaluate the sphere‐forming ability of cell spheroids after WDR72 knockdown. Student's t‐test was used to analyze data of A (left), and one‐way ANOVA was used for A (right). The data of B, C, and E were analyzed via Student's t‐test, while data of F, G and I were analyzed via one‐way ANOVA. ^**^ *p*<0.01.

To further investigate the stemness‐regulating function of WDR72, we overexpressed WDR72 in adherent neuroblastoma cells to determine its impact (Figure 3B). Both RT‐qPCR and Western blot analyses demonstrated that WDR72 overexpression significantly increased the mRNA and protein levels of stemness markers ALDH1 and Nanog (Figure 3C,D). Furthermore, sphere formation assays revealed that WDR72 overexpression markedly enhanced the sphere‐forming capacity of adherent neuroblastoma cells (Figure 3E). These results indicate that WDR72 overexpression induces a stem‐like phenotype in adherent neuroblastoma cells.

Conversely, we knocked down WDR72 in neuroblastoma cell spheroids to examine its impact on stemness (Figure 3F). RT‐qPCR and Western blot analyses showed that WDR72 knockdown significantly reduced the expression of ALDH1 and Nanog (Figure 3G,H). Additionally, sphere formation assays demonstrated that WDR72 knockdown effectively inhibited the sphere‐forming ability of neuroblastoma spheroids (Figure 3I). These findings suggest that WDR72 is essential for maintaining the stemness potential of neuroblastoma spheroids.

In summary, our results collectively demonstrate that WDR72 plays a critical role in enhancing the stemness of neuroblastoma cells, thereby contributing to their malignancy. Through systematic examination of both adherent and spheroid cells, we provide compelling evidence that WDR72 positively regulates stemness in neuroblastoma: overexpression promotes stemness in adherent cells, while knockdown attenuates stemness in spheroids.

### 3.4 The METTL14‐IGF2BP1 Axis Mediated m6A Modification Drives WDR72 Upregulation in Neuroblastoma

To investigate the mechanism underlying the upregulation of WDR72 in neuroblastoma cell spheroids, we first examined the global m6A methylation levels. Dot blot analysis revealed significantly higher methylation levels in neuroblastoma cell spheroids compared to adherent neuroblastoma cells (Figure 4A), with each successive spheroid generation showing a progressive increase in methylation (Figure 4B). This observation suggests that m6A methylation may play a role in WDR72 regulation.

**FIGURE 4:**
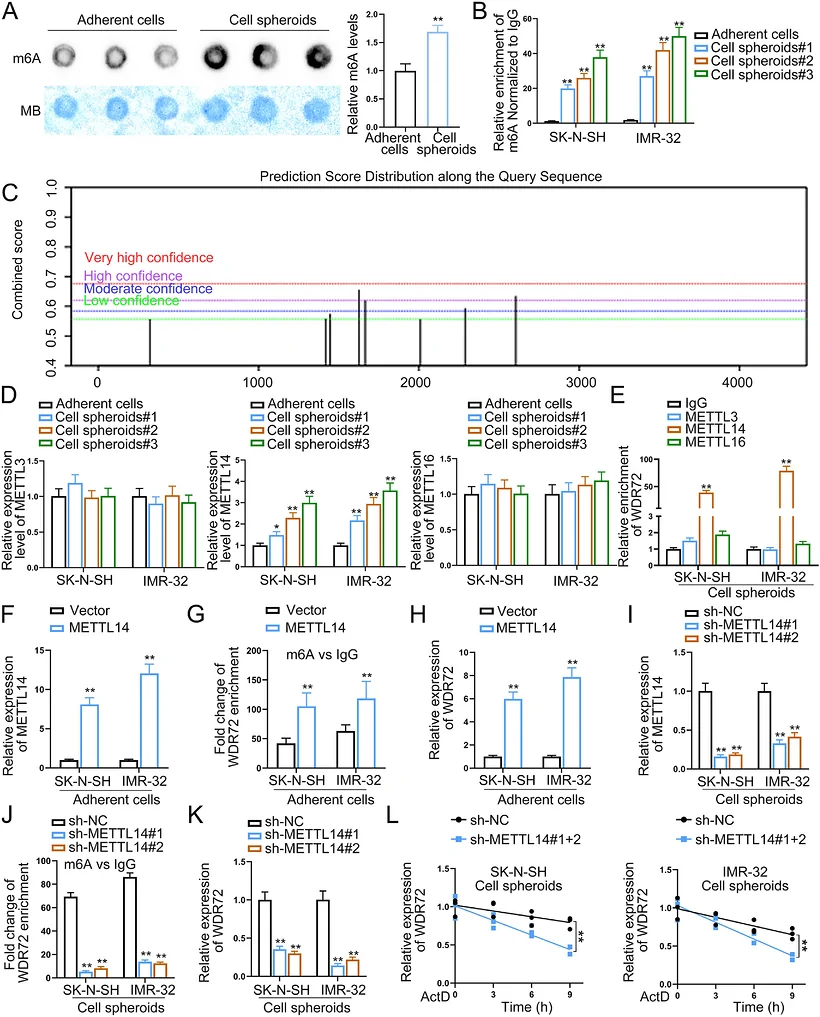
METTL14‐Mediated m6A Modification Induces the Upregulation of WDR72. (A) Dot blot analysis assessed methylation levels in neuroblastoma cell spheroids and adherent neuroblastoma cells. (B) Me‐RIP assays revealed elevated methylation levels in neuroblastoma adherent cells and cell spheroids of different generations. (C) m6A sites in the WDR72 3’UTR were predicted using the SRAMP online bioinformatics analysis tool. (D) The expression of three m6A methyltransferases (METTL3, METTL14, and METTL16) was measured in neuroblastoma adherent cells and cell spheroids by RT‐qPCR. (E) The binding of METTL3, METTL16, and METTL14 to the WDR72 3’UTR was analyzed by RIP assay. (F) METTL14 was overexpressed in neuroblastoma adherent cells, and the overexpression efficiency was detected by RT‐qPCR. (G) Overexpression of METTL14 increased the enrichment of the WDR72 3’UTR in the immunoprecipitates of anti‐m6A, as measured by Me‐RIP assay. (H) RT‐qPCR results indicated that overexpression of METTL14 enhanced WDR72 expression. (I) METTL14 was silenced in neuroblastoma cell spheroids, and knockdown efficiency was detected by RT‐qPCR. (J) The enrichment of the WDR72 3’UTR in the immunoprecipitates of anti‐m6A was measured in METTL14‐silenced neuroblastoma cell spheroids by Me‐RIP assay. (K) The expression level of WDR72 was reduced with the silencing of METTL14, as measured by RT‐qPCR. (L) RT‐qPCR assessed the impact of METTL14 interference on WDR72 expression at various time points. Student's t‐test was used to analyze data of A, F, G and H; one‐way ANOVA was used for B, D, E and I–K, while Two‐way repeated measures ANOVA was used for L. ^**^ *p*<0.01.

To explore this further, we conducted bioinformatics analysis using the SRAMP online tool (http://www.cuilab.cn/sramp) and identified multiple m6A modification sites in the WDR72 3'UTR (Figure 4C). We then assessed the expression of three key m6A methyltransferases (METTL3, METTL14, and METTL16) in neuroblastoma cells and spheroids of different generations. Interestingly, only METTL14 displayed significant upregulation in spheroids, with its expression increasing progressively across generations (Figure 4D and Additional file 1: Figure S4A). RIP assays further confirmed a strong interaction between METTL14 and the WDR72 3’UTR (Figure 4E), indicating that WDR72 is a potential target of METTL14‐mediated m6A modification.

To validate this hypothesis, we overexpressed METTL14 in adherent neuroblastoma cells (Figure 4F), which exhibit low WDR72 expression. Me‐RIP assays demonstrated enhanced m6A enrichment in the WDR72 3’UTR following METTL14 overexpression (Figure 4G), and RT‐qPCR confirmed a corresponding increase in WDR72 mRNA levels (Figure 4H). Conversely, METTL14 knockdown in spheroids, which have high WDR72 expression, led to reduced m6A enrichment and a significant decrease in WDR72 mRNA levels (Figure 4I–K). These results support the role of METTL14 in regulating WDR72 expression through m6A modification.

To comprehensively delineate the m6A‐mediated regulatory network controlling WDR72, we analyzed key m6A erasers (FTO and ALKBH5) (Additional file 1: Figure S4B,C) along with major readers (IGF2BP1, IGF2BP2, and IGF2BP3) (Additional file 1: Figure S4D,E). Strikingly, IGF2BP1 showed pronounced upregulation at both transcriptional and translational levels in neuroblastoma spheroids compared to adherent cultures. These results position IGF2BP1 as a dominant regulatory node for stabilizing m6A‐modified WDR72 mRNA and driving its overexpression. Functional studies using dual‐luciferase reporters confirmed that enforced expression of either METTL14 or IGF2BP1 robustly augmented WDR72 3’UTR activity (Additional file 1: Figure S4F). This epistatic relationship identifies a coordinated METTL14‐IGF2BP1 axis as the central mechanism governing m6A‐dependent WDR72 regulation in spheroid models.

Finally, to assess the impact of m6A modification on WDR72 mRNA stability, we performed RT‐qPCR after METTL14 knockdown. The results revealed a time‐dependent decrease in WDR72 transcripts compared to the control group (Figure 4L), indicating that m6A methylation stabilizes WDR72 mRNA.

Collectively, these findings demonstrate that IGF2BP1‐dependent METTL14‐mediated m6A modification promotes the upregulation of WDR72 in neuroblastoma cell spheroids.

### 3.5 WDR72 Enhances CBX8 Protein Stability to Promote Neuroblastoma Cell Stemness

To investigate the downstream molecular mechanism of WDR72 in neuroblastoma, we performed proteomic analysis to identify proteins regulated by WDR72 (Figure 5A). Among the candidates, CBX8, an oncogene which was highly suggested to be associated with cancer stemness [19, 20, 21], was focused on since it exhibited the most significant upregulation upon WDR72 overexpression. We validated the positive regulatory effect of WDR72 on CBX8 protein through Western blot analysis, observing decreased CBX8 protein levels in WDR72‐silenced neuroblastoma cells (Figure 5B). Furthermore, we examined CBX8 expression in metastatic tissues to determine its correlation with high WDR72 levels. Both protein and mRNA analyses, along with tissue microarray data, revealed a significant upregulation of CBX8 in metastatic tissues (Additional file 1: Figure S5A–C), with a notable correlation between CBX8 and WDR72 protein levels (Additional file 1: Figure S5D), suggesting a potential role for CBX8 in the malignancy of neuroblastoma.

**FIGURE 5:**
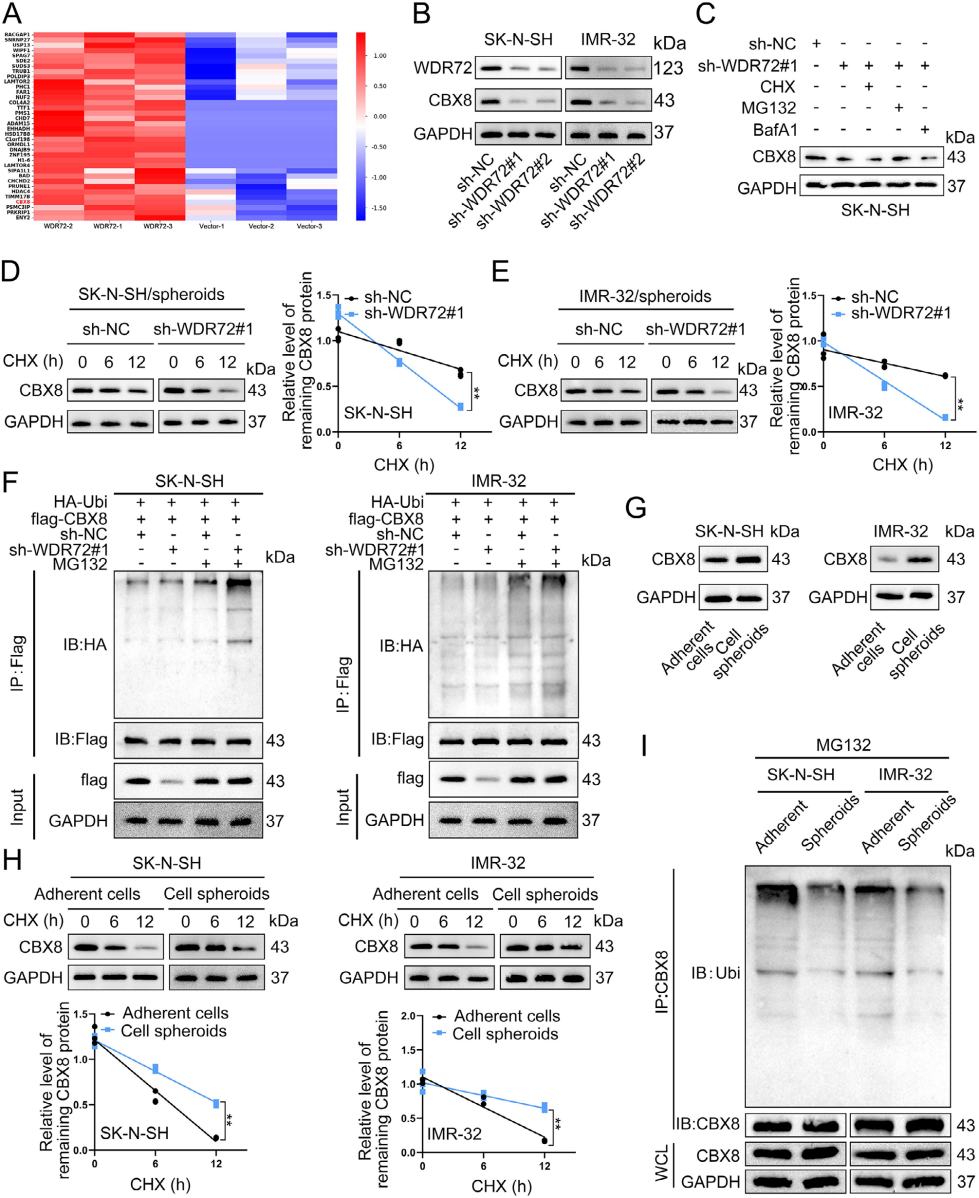
WDR72 Enhances the Stability of CBX8 Protein. (A) The downstream proteins of WDR72 in IMR‐32 cells transfected with WDR72 overexpression vector or control vector were analyzed by proteomic analysis. (B) Western blot detection of WDR72 and CBX8 expression after WDR72 knockdown. (C) Treatment of cells with WDR72 inhibitors, CHX, MG132, or BafA1, followed by Western blot analysis of CBX8 expression changes. (D,E) Western blot analysis of the effect of WDR72 knockdown on CBX8 degradation after inhibition of protein synthesis with CHX. (F) Co‐IP experiment to analyze the ubiquitination level of CBX8 after WDR72 knockdown. (G) Western blot detection of the differential expression of CBX8 in adherent cells and cell spheroids. (H) Western blot analysis of the changes in CBX8 levels in adherent cells and cell spheroids at different time points after inhibition of protein synthesis with CHX. (I) Co‐IP experiment to detect the ubiquitination level of CBX8 in adherent cells and cell spheroids after inhibition of protein degradation. Two‐way repeated measures ANOVA was used to analyze data of D, E and H. ^**^ *p*<0.01.

CBX8 has been implicated in the formation of the cancer stem cell phenotype [20]. To assess its functional role in neuroblastoma, we performed sphere formation and colony formation assays. Overexpression of CBX8 in adherent cells promoted sphere formation and enhanced cell proliferation capacity (Additional file 1: Figure S5E). Oppositely, CBX8 knockdown suppressed cell proliferation and sphere formation, while such suppressive effects were normalized under CBX8 recovery via co‐transfecting Flag‐CBX8‐loaded plasmids (Additional file 1: Figure S5F,G). These findings indicate that CBX8 promotes oncogenic effects, and the regulatory relationship between WDR72 and CBX8 suggests that WDR72 may exert its oncogenic effects partially through CBX8.

To further elucidate the mechanism by which WDR72 regulates CBX8, we treated neuroblastoma cells with cycloheximide (CHX, a protein synthesis inhibitor), MG132 (a proteasome inhibitor), or bafilomycin A1 (BafA1, an autophagy‐lysosome inhibitor) after WDR72 knockdown. WDR72 silencing did not alter CBX8 protein levels in MG132‐treated cells, indicating that WDR72 likely regulates CBX8 degradation (Figure 5C). Consistent with this, WDR72 knockdown shortened the half‐life of CBX8 protein in CHX‐treated neuroblastoma cells (Figures 5D‐E), while WDR72 overexpression extended the half‐life of CBX8 protein and increased its expression levels (Additional file 1: Figure S6A,B). The ubiquitin‐proteasome system plays a pivotal role in protein degradation, and our Co‐IP and Western blot analyses revealed that WDR72 knockdown increased CBX8 ubiquitination (Figure 5F), suggesting that WDR72 stabilizes CBX8 by reducing its ubiquitination. Notably, CBX8 protein levels were significantly higher in neuroblastoma cell spheroids compared to adherent neuroblastoma cells (Figure 5G). Furthermore, the half‐life of CBX8 protein was longer in neuroblastoma spheroids (Figure 5H), and its ubiquitination levels were lower in these neuroblastoma cells (Figure 5I). These results demonstrate that WDR72 enhances CBX8 stability through de‐ubiquitination.

To directly assess the functional role of the WDR72‐CBX8 axis in neuroblastoma cell stemness, we performed rescue experiments. Overexpression of CBX8 in WDR72‐knockdown neuroblastoma cells significantly restored colony formation and sphere formation capabilities (Additional file 1: Figure S6C,D). Likewise, the enhancement of WDR72 overexpression on neuroblastoma cell proliferation and sphere formation was abrogated after CBX8 knockdown (Additional file 1: Figure S6E,F). These data demonstrated that CBX8 acts as a critical downstream effector of WDR72 in maintaining CSC properties. This functional rescue provides strong evidence supporting the mechanistic link between WDR72 and CBX8, confirming their combined role in promoting neuroblastoma cell stemness.

Together, these findings elucidate a novel regulatory mechanism in which WDR72 stabilizes CBX8 protein through de‐ubiquitination, thereby enhancing neuroblastoma cell stemness.

### 3.6 WDR72 Inhibits TRIM31‐Mediated Ubiquitination of CBX8 to Enhance Its Stability

Our previous findings suggested that WDR72 mediates the de‐ubiquitination of CBX8, enhancing its stability. To explore the underlying mechanism, we first performed an IP assay to determine whether WDR72 directly regulates CBX8. However, no direct interaction was observed between the two proteins (Additional file 1: Figure S7A).

Next, we identified potential CBX8 interactors through IP and mass spectrometry analysis (Figure 6A). Among the candidates, TRIM31, a newly identified E3 ubiquitin ligase of the TRIM family involved in autophagy, inflammation, antiviral immunity, and cancer, caught our attention. We confirmed the interaction between TRIM31 and CBX8 using Co‐IP and Western blot analyses (Figure 6B), suggesting TRIM31's potential regulatory role. Functional assays revealed that TRIM31 interference enhanced proliferation, sphere‐forming ability, and CBX8 activity in neuroblastoma cells (Additional file 1: Figure S7B–D), indicating its negative regulatory role on CBX8.

**FIGURE 6:**
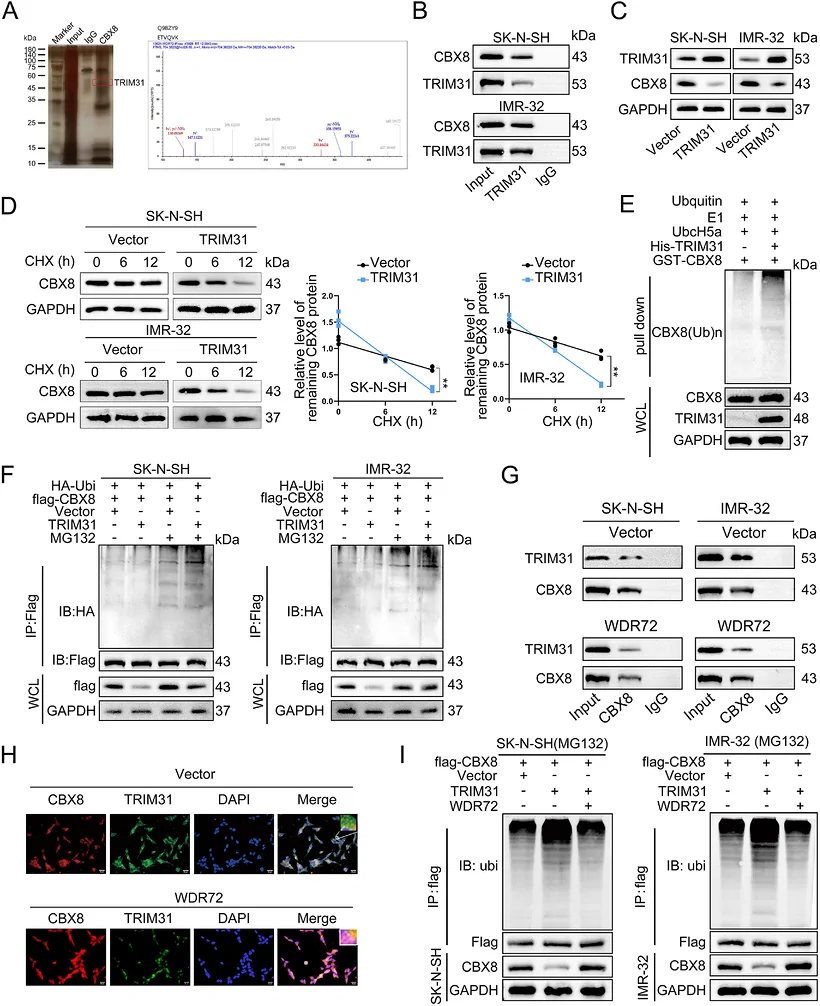
WDR72 Inhibits TRIM31‐Mediated Ubiquitination of CBX8 to Enhance Its Stability. (A) TRIM31 protein was screened using IP‐MS analysis. (B) Co‐IP was performed to detect the interaction between CBX8 and TRIM31. (C) Western blot analysis was conducted to investigate the regulatory effect of TRIM31 overexpression on CBX8 expression. (D) Analysis of CBX8 degradation after TRIM31 overexpression in the presence of CHX to inhibit protein synthesis. (E) GST pull down assay to determine if CBX8 is a substrate of TRIM31. (F) Co‐IP experiment to analyze the ubiquitination level of CBX8 and the effect of TRIM31 overexpression on CBX8 ubiquitination. (G) Co‐IP experiment to examine the impact of WDR72 overexpression on the interaction between TRIM31 and CBX8. (H) IF co‐localization experiment to analyze the effect of WDR72 overexpression on the binding of TRIM31 and CBX8. (I) In vitro ubiquitination assay to determine the impact of TRIM31 and WDR72 on CBX8 ubiquitination. Two‐way repeated measures ANOVA was used to analyze data of D. ^**^ *p*<0.01.

To further elucidate the mechanism, we overexpressed TRIM31 in neuroblastoma cells and observed a reduction in CBX8 protein levels (Figure 6C). Western blot analysis showed that TRIM31 overexpression shortened the half‐life of CBX8 (Figure 6D). GST pull down assays confirmed that CBX8 is a substrate of TRIM31 (Figure 6E), and TRIM31 overexpression significantly increased CBX8 ubiquitination both exogenously and endogenously (Figure 6F and Additional file 1: Figure S8A), demonstrating TRIM31's role in promoting CBX8 ubiquitination.

Given the opposing roles of WDR72 and TRIM31 in CBX8 ubiquitination, we investigated their potential interaction. While WDR72 overexpression did not alter TRIM31 levels (Additional file 1: Figure S8B), Co‐IP assays confirmed interactions between WDR72 or CBX8 and TRIM31 (Additional file 1: Figure S8C). WDR72 overexpression disrupted the TRIM31‐CBX8 interaction in adherent cells (Figure 6G and Additional file 1: Figure S8D), whereas WDR72 interference enhanced this interaction in spheroids (Additional file 1: Figure S8E). Immunofluorescence assays further validated the WDR72‐TRIM31 interaction and its impact on TRIM31‐CBX8 binding (Figure 6H and Additional file 1: Figure S8F). Ubiquitination assays demonstrated that WDR72 effectively reduces TRIM31‐induced CBX8 ubiquitination (Figure 6I).

Notably, we observed lower TRIM31 expression in metastatic neuroblastoma tissues (Additional file 1: Figure S9A–C). Meanwhile, we also detected the staining of CBX8 in the same tissues used in Figure S9C, and the data showed that CBX8 staining was elevated in metastatic neuroblastoma tissues compared to non‐metastatic controls (Additional file 1: Figure S9D). More importantly, the staining of CBX8 in these samples was negatively correlated with that of TRIM31 (Additional file 1: Figure S9E), further supporting that CBX8 was inhibited by TRIM31‐mediated ubiquitination. In summary, our findings indicate that WDR72 impedes the TRIM31‐CBX8 interaction, thereby stabilizing CBX8 protein.

### 3.7 WDR72 Stabilizes and Activates CBX8 by Inducing K63‐linked Ubiquitination and Repressing K48‐linked Ubiquitination Through Interaction With TRIM31

The type of polyubiquitination chain plays a crucial role in determining the fate of proteins [22]. After confirming that TRIM31 mediates CBX8 ubiquitination, we sought to identify the specific type of ubiquitination involved. Using Co‐IP and Western blot analyses, we found TRIM31 overexpression significantly reduced K63‐linked ubiquitination while increasing K48‐linked ubiquitination of CBX8 (Figure 7A). Conversely, TRIM31 interference decreased K48‐linked ubiquitination of CBX8 (Figure 7B). Importantly, these effects were abolished when both K48 and K63 ubiquitin chains were mutated (Figure 7C). Furthermore, the TRIM31 C36A mutant, which lacks E3 ligase activity, failed to inhibit K63‐linked ubiquitination and also enhanced K48‐linked ubiquitination of CBX8 (Figure 7D,E), indicating that TRIM31's E3 ligase activity is essential for modulating these ubiquitination patterns.

**FIGURE 7:**
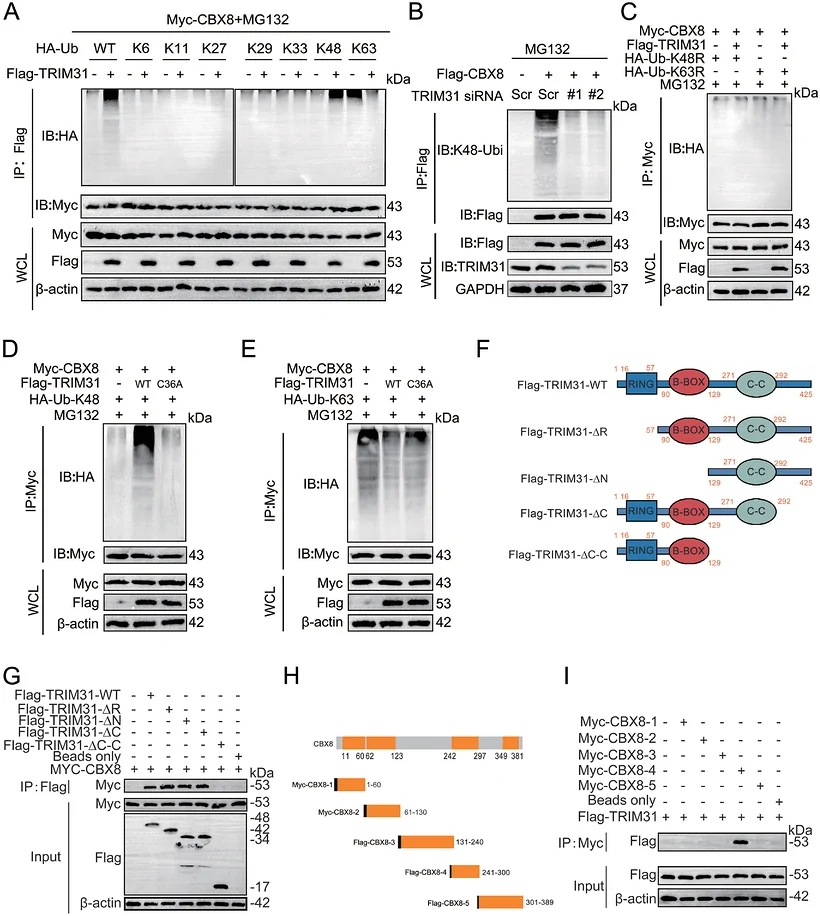
WDR72 Stabilizes and Activates CBX8 by Inducing K63‐linked Ubiquitination and Repressing K48‐Linked Ubiquitination through Interaction with TRIM31. (A) Construction of ubiquitin chain‐tagged vectors for Co‐IP experiment to elucidate the specific ubiquitin chain mediated by TRIM31 on CBX8. (B) Co‐IP experiment to analyze the impact of TRIM31 knockdown on the ubiquitination level of the CBX8 K48 chain. (C) Co‐IP experiment after mutating K63 and K48 to analyze the role of TRIM31. (D,E) Analysis of the changes in the roles of TRIM31‐mediated K63 and K48 ubiquitination after mutating the E3 ubiquitin ligase active site of TRIM31 (C36A). (F) Schematic representation of TRIM31's domain constructs. (G) Co‐IP experiment to analyze the binding region between TRIM31 and CBX8. (H) Schematic representation of CBX8's domain constructs. (I) Co‐IP experiment to analyze the binding region between CBX8 and TRIM31. ^**^ *p*<0.01.

Immunofluorescence co‐localization assays revealed that WDR72 overexpression in adherent cells or knockdown in spheroid cells altered the levels of K48‐ and K63‐linked ubiquitination of CBX8. Specifically, WDR72 overexpression increased K63‐linked ubiquitination while decreasing K48‐linked ubiquitination (Additional file 1: Figure S10A), whereas, WDR72 knockdown had the opposite effect (Additional file 1: Figure S10B). These findings strongly suggest that TRIM31 promotes K48‐linked ubiquitination of CBX8 via its E3 ligase activity, a process regulated by WDR72.

TRIM31 comprises three functional domains: a RING‐finger domain (amino acids 16–57), a B‐box domain (amino acids 90–129), and a C‐C domain (amino acids 126–307) [23]. To identify the domain responsible of TRIM31's interaction with CBX8, we generated truncation mutants of TRIM31 (Figure 7F). Co‐IP experiments showed that TRIM31‐WT, TRIM31‐∆R, TRIM31‐∆N, and TRIM31‐∆C mutants retained their ability to interact with CBX8, while the Flag‐TRIM31‐∆C‐C mutant and the control (beads alone) did not (Figure 7G). Western blot, clone formation and sphere formation assays, demonstrated that the deletion of the CC domain (∆C‐C) or the disruption of the RING domain (either ∆R deletion or C36A mutation) in TRIM31 abolished its ability to suppress CBX8 activity (Additional file 1: Figure S11A–C).

To further delineate the interaction domains within CBX8, we divided CBX8 into five segments based on its structural domains and constructed tagged vectors (Figure 7H): Myc‐CBX8‐1 (amino acids 1–60), Myc‐CBX8‐2 (amino acids 61–300), Myc‐CBX8‐3 (amino acids 131–240), Myc‐CBX8‐4 (amino acids 241–300), and Myc‐CBX8‐5 (amino acids 301–389). Co‐IP assays revealed that TRIM31 specifically interacts with the Myc‐CBX8‐4 segment (amino acids 241–300) (Figure 7I).

In summary, our results demonstrate that WDR72 stabilizes and activates CBX8 by promoting K63‐linked ubiquitination and suppressing K48‐linked ubiquitination through its interaction with TRIM31. This regulatory mechanism is mediated by specific domains within TRIM31 and CBX8, highlighting the intricate interplay between these proteins in modulating CBX8 stability and activity.

### 3.8 K48 And K63 Ubiquitin Chains Target K227 and K295 Sites of CBX8 Respectively, and Cooperatively Regulate Its Ubiquitination

To precisely map the ubiquitination sites associated with K48‐ and K63‐linked polyubiquitin chains on CBX8, we conducted bioinformatics analysis, which identified four potential ubiquitination sites (Figure 8A). Using site‐directed mutagenesis, we generated mutant CBX8 constructs and performed immunoprecipitation assays. Our results revealed that the K227R mutation specifically impaired K48‐linked ubiquitination, while the K295R mutation disrupted K63‐linked ubiquitination of CBX8 (Figure 8B).

**FIGURE 8:**
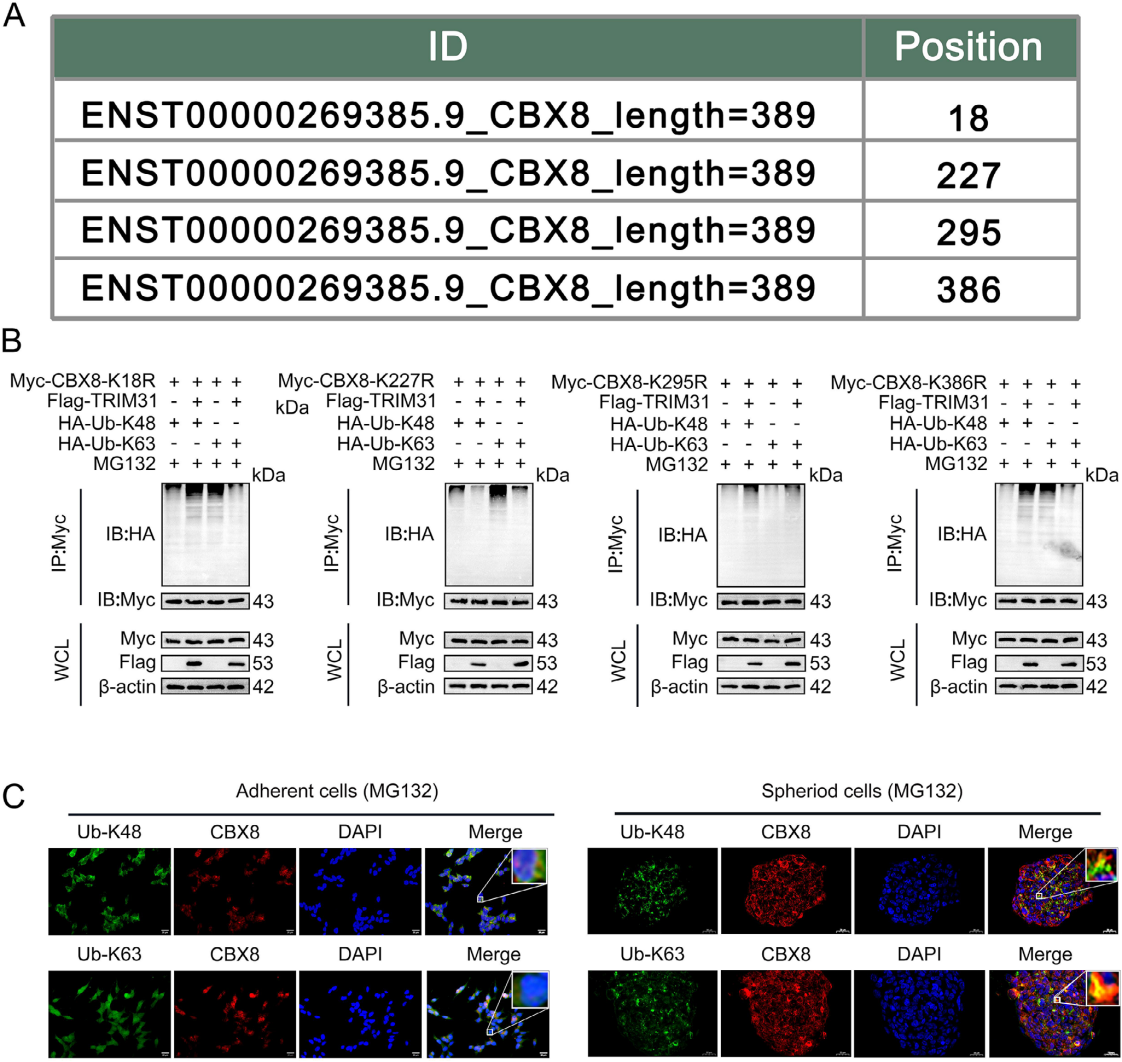
K48 and K63 ubiquitin chains target K227 and K295 sites of CBX8 respectively, and cooperatively regulate its ubiquitination. (A) Bioinformatics website screening for ubiquitination sites of CBX8. (B) Co‐IP experiment to screen for ubiquitination sites. (C) Immunofluorescence analysis of ubiquitin chains on CBX8 in adherent cells and cell spheroids after treatment with MG132.

To further characterize the ubiquitination patterns of CBX8 under different cellular conditions, we treated cells with the proteasome inhibitor MG132 and analyzed ubiquitin chain modifications in both adherent and sphere‐forming cells. Notably, K48‐linked ubiquitination was predominantly observed in adherent cells, whereas K63‐linked ubiquitination was significantly enriched in sphere‐forming cells (Figure 8C).

In functional assays, including colony formation and sphere formation, we found that overexpression of TRIM31 inhibited the promoting effects of CBX8 on the colony formation and sphere formation capabilities of neuroblastoma cells. Additionally, transfection with either the CBX8‐K295R mutant or the CBX8‐K227R mutant weakened the degradation effect of TRIM31 on CBX8, with the CBX8‐K227R mutant exhibiting a stronger inhibitory effect. Importantly, simultaneous mutations at both sites (K227R + K295R) made it resistant to TRIM31‐mediated degradation, restoring CBX8 function (Additional file 1: Figure S11D,E).

## 4 Discussion

Cancer stem cells (CSCs) are key drivers of tumor progression, recurrence, and therapeutic resistance, playing a central role in tumor malignancy [24, 25]. In this study, we identified WDR72 as a CSC marker associated with high‐risk neuroblastoma and poor prognosis through integrated analysis of the TARGET‐NBL and GEO databases. Importantly, our work is the first to systematically elucidate the functional roles and regulatory mechanisms of WDR72 in neuroblastoma stemness and progression, providing novel insights into its contribution to neuroblastoma pathology.

Previous studies have implicated WDR72 in various cancer types. For instance, WDR72 has been proposed as a biomarker for predicting recurrence in bladder cancer [26], aiding in the diagnosis of esophageal cancer [27], and functioning as a tumor suppressor linked to prognosis in renal cell carcinoma [28]. Our findings expand on these observations by demonstrating that WDR72 silencing significantly suppresses neuroblastoma cell viability and proliferation while markedly increasing apoptosis. Furthermore, WDR72 knockdown impaired the migratory and invasive capabilities of neuroblastoma cells, establishing a strong positive correlation between WDR72 expression and neuroblastoma cell malignancy.

A pivotal aspect of our study is the exploration of WDR72's role in regulation neuroblastoma cell stemness. We observed that WDR72 expression is significantly upregulated in neuroblastoma cell spheroids compared to adherent cells, a finding consistent with the enhanced stemness potential of spheroids. Functional assays revealed that WDR72 overexpression increases the expression of stemness markers such as ALDH1 and Nanog, while also promoting the sphere‐forming capacity of both adherent cells and spheroids. These results collectively demonstrate that WDR72 is a critical enhancer of neuroblastoma cell stemness, which has been previously suggested in lung cancer as well [10]. Also, the finding is consistent with previous report that proposed WDR72 as CSC‐related genes in gastric cancer [12].

To unravel the mechanism underlying WDR72 upregulation in neuroblastoma cell spheroids, we employed bioinformatics analysis and identified multiple m6A modification sites in the WDR72 3’UTR. m6A modification is a well‐characterized post‐transcriptional regulatory mechanism that influences mRNA stability and translation, thereby impacting tumor progression. For example, METTL3‐mediated m6A modification of HDGF mRNA facilitates gastric cancer progression [29], while IGF2BP2‐dependent m6A modification of HMGA1 drives colorectal cancer proliferation and metastasis [30]. Our m6A‐RIP assays confirmed m6A modification of WDR72, with higher m6A enrichment observed in the WDR72 3’UTR of neuroblastoma cell spheroids. Among the m6A writers (METTL3, METTL14, and METTL16) and the readers (IGF2BP1, IGF2BP2, and IGF2BP3) [31, 32, 33, 34, 35, 36], we found that METTL14 and IGF2BP1 were significantly upregulated in neuroblastoma cell spheroids and could directly bind to the WDR72 3’UTR. These findings reveal for the first time that the METTL14‐IGF2BP1 axis mediates m6A modifications that drive the upregulation of WDR72 in neuroblastoma cell spheroids.

Beyond m6A regulation, we investigated the downstream target of WDR72 and their roles in neuroblastoma cell stemness. Proteins often exert their oncogenic effects by modulating downstream target [37, 38, 39]. Through proteomic analysis, we identified CBX8 as a key effector of WDR72 in neuroblastoma. Mechanistic studies revealed that WDR72 stabilizes CBX8 protein by reducing its ubiquitination levels. CBX8 has been implicated as potential diagnostic and prognostic biomarker for several common cancers including hepatocellular carcinoma, kidney renal clear cell carcinoma, and ovarian cancer [40]. Also, it has been proved as a tumor promoter in colon cancer [41, 42], and laryngeal squamous cell carcinoma [43], and lung cancer [44]. More importantly, CBX8 is suggested to be highly associated with cancer stemness as well [19]. However, our study is the first to link CBX8 to neuroblastoma stemness. Since WDR72 does not directly interact with CBX8, we further identified TRIM31, an E3 ubiquitin ligase [23], as a CBX8‐interacting partner. Our results demonstrate that TRIM31 promotes CBX8 ubiquitination, while WDR72 inhibits the TRIM31‐CBX8 interaction, thereby stabilizing CBX8 protein. This discovery underscores the role of WDR72 in modulating CBX8 stability through a TRIM31‐dependent mechanism.

Our study demonstrates that WDR72 promotes neuroblastoma stemness and progression through a multi‐layered regulatory mechanism. First, WDR72 is upregulated through m6A modifications mediated by the METTL14‐IGF2BP1 axis, thereby enhancing the specific stability of its mRNA in neuroblastoma cell spheroids. Second, WDR72 stabilizes CBX8 by disrupting its interaction with the E3 ubiquitin ligase TRIM31, thereby inhibiting TRIM31‐mediated ubiquitination and subsequent proteasomal degradation. These findings elucidate a previously unrecognized molecular axis driving neuroblastoma malignancy and highlight WDR72 as a potential therapeutic target for future interventions in neuroblastoma treatment.

## Author Contributions

**Yan Zou**, **Huijuan Zeng** and **Tianyou Yang** designed the experiments. **Huijuan Zeng**, **Zijie Ye**, and **Manna Zheng** collected the clinical samples, performed experiment and analyzed the patients’ information. **Jing Pan** and **Tianbao Tan** performed the animal experiment. **Jiliang Yang**, **Jiahao Li**, and **Zhang Zhao** performed IHC and some animal experiment. **Tianzhu Long** and **Liyu Zhang** supervised the analysis. **Chao Hu** drafted the initial manuscript. **Gautam Sethi**, Tianyou Yang and Yan Zou revised the manuscript. All authors have read and approved the final manuscript.

## Ethics Statement

The procedures of this study were approved by the Research Ethics Committee of Guangzhou Women and Children's Medical Center (No.[2023]064B00) and conducted in accordance with the Code of Ethics of the World Medical Association. The experimental methods comply with the Helsinki Declaration. All experiments were performed in accordance with the Guide for the Care and Use of Laboratory Animals, and authorized by Guangzhou Women and Children's Medical Center (No. G20230376).

## Conflicts of Interest

The authors declare no conflicts of interest.

## Supporting information




**Supporting File 1**: advs76602‐sup‐0001‐SuppMat.docx.

**Supporting File 2**: advs76602‐sup‐0002‐FigureS1‐S11.zip

**Supporting File 3**: advs76602‐sup‐0003‐TableS1.xlsx.

## Data Availability

The data that support the findings of this study are available from the corresponding author upon reasonable request.

## References

[1] R. Sharma , J. Yadav , S. A. Bhat , et al., “Emerging Trends in Neuroblastoma Diagnosis, Therapeutics, and Research,” Molecular Neurobiology 62 (2025): 6423–6466. 10.1007/s12035-024-04680-w 39804528

[2] L. Sainero‐Alcolado , T. Sjöberg Bexelius , G. Santopolo , Y. Yuan , J. Liaño‐Pons , and M. Arsenian‐Henriksson , “Defining Neuroblastoma: From Origin to Precision Medicine,” Neuro‐Oncology 26 (2024): 2174–2192. 10.1093/neuonc/noae152 PMC11630532 39101440

[3] J. Stankiewicz , M. Pogorzała , P. Księżniakiewicz , and J. Styczyński , “From Local to International Approach: Prognostic Factors and Treatment Outcomes in Neuroblastoma‐A 30‐Year Single‐Center Retrospective Analysis,” Children (Basel) 12 (2025): 525. 10.3390/children12040525 PMC12025632 40310212

[4] N. H. Freire , M. D. C. Jaeger , C. B. de Farias , et al., “Targeting the Epigenome of Cancer Stem Cells in Pediatric Nervous System Tumors,” Molecular and Cellular Biochemistry 478 (2023): 2241–2255. 10.1007/s11010-022-04655-2 36637615

[5] P. A. Riya , B. Basu , S. Surya , et al., “HES1 Promoter Activation Dynamics Reveal the Plasticity, Stemness and Heterogeneity in Neuroblastoma Cancer Stem Cells,” Journal of Cell Science 135 (2022): jcs.260157. 10.1242/jcs.260157 36321463

[6] S. Krishna , B. Prajapati , P. Seth , and S. Sinha , “Dickopff 1 Inhibits Cancer Stem Cell Properties and Promotes Neuronal Differentiation of Human Neuroblastoma Cell Line SH‐SY5Y,” IBRO Neuroscience Reports 17 (2024): 73–82. 10.1016/j.ibneur.2024.05.010 PMC11253693 39021664

[7] G. Shi , Q. Ni , Y. Miao , et al., “Identification of WD‐Repeat Protein 72 as a Novel Prognostic Biomarker in Non‐Small‐Cell Lung Cancer,” Mediators of Inflammation 2023 (2023): 2763168. 10.1155/2023/2763168 PMC10185422 37197572

[8] H. Zhang , M. Koruyucu , F. Seymen , et al., “WDR72 Mutations Associated with Amelogenesis Imperfecta and Acidosis,” Journal of Dental Research 98 (2019): 541–548. 10.1177/0022034518824571 PMC6481005 30779877

[9] K. Katsura , Y. Nakano , Y. Zhang , R. Shemirani , W. Li , and P. D. Besten , “WDR72 Regulates Vesicle Trafficking in Ameloblasts,” Scientific Reports 12 (2022): 2820. 10.1038/s41598-022-06751-1 PMC8857301 35181734

[10] X. Ouyang , X. Shi , N. Huang , et al., “WDR72 Enhances the Stemness of Lung Cancer Cells by Activating the AKT/HIF‐1α Signaling Pathway,” Journal of Oncology 2022 (2022): 5059588. 10.1155/2022/5059588 PMC9663245 36385964

[11] G. Shi , J. Wei , S. Rahemu , J. Zhou , and X. Li , “Study on the Regulatory Mechanism of Luteolin Inhibiting WDR72 on the Proliferation and Metastasis of Non Small Cell lung Cancer,” Scientific Reports 15 (2025): 12398. 10.1038/s41598-025-96666-4 PMC11992086 40216870

[12] L. Zheng , J. Lu , D. Kong , and Y. Zhan , “Single‐Cell Sequencing Analysis Revealed That WDR72 was a Novel Cancer Stem Cells Related Gene in Gastric Cancer,” Heliyon 10 (2024): 35549. 10.1016/j.heliyon.2024.e35549 PMC11336769 39170171

[13] F. Pastorino , C. Brignole , D. Marimpietri , et al., “Vascular Damage and Anti‐angiogenic Effects of Tumor Vessel‐Targeted Liposomal Chemotherapy,” Cancer research 63 (2003): 7400–7409. 14612539

[14] B. Brazvan , R. Farahzadi , S. M. Mohammadi , et al., “Key Immune Cell Cytokines Affects the Telomere Activity of Cord Blood Cells In vitro,” Advanced Pharmaceutical Bulletin 6 (2016): 153–161. 10.15171/apb.2016.022 PMC4961972 27478776

[15] C. A. Formolo , R. Williams , H. Gordish‐Dressman , T. J. MacDonald , N. H. Lee , and Y. Hathout , “Secretome Signature of Invasive Glioblastoma Multiforme,” Journal of Proteome Research 10 (2011): 3149–3159. 10.1021/pr200210w PMC3136381 21574646

[16] H. Zeng , C. Hu , M. Zheng , et al., “WDR72 promotes neuroblastoma stemness and progression by sequestering TRIM31‐mediated degradation of CBX8” OMIX (2025), https://ngdc.cncb.ac.cn/search/specific?db=omix&q=OMIX010833. 10.1002/advs.76602 PMC13423489 42531598

[17] H. Zeng , C. Hu , M. Zheng , et al., “WDR72 promotes neuroblastoma stemness and progression by sequestering TRIM31‐mediated degradation of CBX8,” OMIX (2025), https://ngdc.cncb.ac.cn/search/specific?db=omix&q=OMIX010974. 10.1002/advs.76602 PMC13423489 42531598

[18] Y. Chen , C. Peng , J. Chen , et al., “WTAP Facilitates Progression of Hepatocellular Carcinoma via m6A‐HuR‐Dependent Epigenetic Silencing of ETS1,” Molecular Cancer 18 (2019): 127. 10.1186/s12943-019-1053-8 PMC6704583 31438961

[19] L. Ng , H. S. Li , A. T. Man , et al., “High Expression of a Cancer Stemness‐Related Gene, Chromobox 8 (CBX8), in Normal Tissue Adjacent to the Tumor (NAT) Is Associated With Poor Prognosis of Colorectal Cancer Patients,” Cells 11 (2022): 1852. 10.3390/cells11111852 PMC9180723 35681547

[20] B. Tang , Y. Tian , Y. Liao , et al., “CBX8 Exhibits Oncogenic Properties and Serves as a Prognostic Factor in Hepatocellular Carcinoma,” Cell Death & Disease 10 (2019): 52. 10.1038/s41419-018-1288-0 PMC6361915 30718464

[21] A. J. van Wijnen , L. Bagheri , A. A. Badreldin , et al., “Biological Functions of Chromobox (CBX) Proteins in Stem Cell Self‐Renewal, Lineage‐Commitment, Cancer and Development,” Bone 143 (2021): 115659. 10.1016/j.bone.2020.115659 32979540

[22] J. Liu , Y. Cheng , M. Zheng , et al., “Targeting the Ubiquitination/Deubiquitination Process to Regulate Immune Checkpoint Pathways,” Signal Transduction and Targeted Therapy 6 (2021): 28. 10.1038/s41392-020-00418-x PMC7819986 33479196

[23] Y. Guo , Q. Li , G. Zhao , et al., “Loss of TRIM31 Promotes Breast Cancer Progression Through Regulating K48‐ and K63‐Linked Ubiquitination of p53,” Cell Death & Disease 12 (2021): 945. 10.1038/s41419-021-04208-3 PMC8516922 34650049

[24] K. Qureshi‐Baig , D. Kuhn , E. Viry , et al., “Hypoxia‐Induced Autophagy Drives Colorectal Cancer Initiation and Progression by Activating the PRKC/PKC‐EZR (ezrin) Pathway,” Autophagy 16 (2020): 1436–1452. 10.1080/15548627.2019.1687213 PMC7469473 31775562

[25] L. Bi , Y. Ren , M. Feng , et al., “HDAC11 Regulates Glycolysis Through the LKB1/AMPK Signaling Pathway to Maintain Hepatocellular Carcinoma Stemness,” Cancer Research 81 (2015): 2015–2028. 10.1158/0008-5472.CAN-20-3044 33602787

[26] J. Mares , M. Szakacsova , V. Soukup , J. Duskova , A. Horinek , and M. Babjuk , “Prediction of Recurrence in Low and Intermediate Risk Non‐Muscle Invasive Bladder Cancer by Real‐Time Quantitative PCR Analysis: CDNA Microarray Results,” Neoplasma 60 (2013): 295–301. 10.4149/neo_2013_0391 23452234

[27] U. Warnecke‐Eberz , R. Metzger , A. H. Hölscher , U. Drebber , and E. Bollschweiler , “Diagnostic Marker Signature for Esophageal Cancer From Transcriptome Analysis,” Tumor Biology 37 (2016): 6349–6358. 10.1007/s13277-015-4400-4 26631031

[28] Y. Zou , Q. Lu , Q. Yao , D. Dong , and B. Chen , “Identification of Novel Prognostic Biomarkers in Renal Cell Carcinoma,” Aging 12 (2020): 25304–25318. 10.18632/aging.104131 PMC7803519 33234734

[29] Q. Wang , C. Chen , Q. Ding , et al., “METTL3‐Mediated m 6 A Modification of HDGF mRNA Promotes Gastric Cancer Progression and has Prognostic Significance,” Gut 69 (2020): 1193–1205. 10.1136/gutjnl-2019-319639 31582403

[30] P. Hou , S. Meng , M. Li , et al., “LINC00460/DHX9/IGF2BP2 Complex Promotes Colorectal Cancer Proliferation and Metastasis by Mediating HMGA1 mRNA Stability Depending on m6A Modification,” Journal of Experimental & Clinical Cancer Research 40 (2021): 52. 10.1186/s13046-021-01857-2 PMC7851923 33526059

[31] H. Li , C. Wang , L. Lan , et al., “METTL3 Promotes Oxaliplatin Resistance of Gastric Cancer CD133+ Stem Cells by Promoting PARP1 mRNA Stability,” Cellular and Molecular Life Sciences 79 (2022): 135. 10.1007/s00018-022-04129-0 PMC11072755 35179655

[32] J. Chen , H. Zhang , C. Xiu , et al., “METTL3 Promotes Pancreatic Cancer Proliferation and Stemness by Increasing Stability of ID2 mRNA in a m6A‐Dependent Manner,” Cancer Letters 565 (2023): 216222. 10.1016/j.canlet.2023.216222 37196908

[33] F. Peng , J. Xu , B. Cui , et al., “Oncogenic AURKA‐Enhanced N6‐Methyladenosine Modification Increases DROSHA mRNA Stability to Transactivate STC1 in Breast Cancer Stem‐Like Cells,” Cell Research 31 (2021): 345–361. 10.1038/s41422-020-00397-2 PMC8027457 32859993

[34] H. Wang , W. Wei , Z. Y. Zhang , et al., “TCF4 and HuR Mediated‐METTL14 Suppresses Dissemination of Colorectal Cancer via N6‐Methyladenosine‐Dependent Silencing of ARRDC4,” Cell Death & Disease 13 (2021): 3. 10.1038/s41419-021-04459-0 PMC8677753 34916487

[35] X. K. Wang , Y. W. Zhang , C. M. Wang , et al., “METTL16 Promotes Cell Proliferation by Up‐Regulating Cyclin D1 Expression in Gastric Cancer,” Journal of Cellular and Molecular Medicine 25 (2021): 6602–6617. 10.1111/jcmm.16664 PMC8278090 34075693

[36] W. Guo , Q. Huai , H. Wan , et al., “Prognostic Impact of IGF2BP3 Expression in Patients With Surgically Resected Lung Adenocarcinoma,” DNA and Cell Biology 40 (2021): 316–331. 10.1089/dna.2020.6136 33493403

[37] A. Al‐Eidan , Y. Wang , P. Skipp , and R. M. Ewing , “The USP7 Protein Interaction Network and Its Roles in Tumorigenesis,” Genes & Diseases 9 (2022): 41–50. 10.1016/j.gendis.2020.10.004 PMC8720671 35005106

[38] M. Słabicki , Z. Kozicka , G. Petzold , et al., “The CDK Inhibitor CR8 Acts as a Molecular Glue Degrader that Depletes Cyclin K,” Nature 585 (2020): 293–297. 10.1038/s41586-020-2374-x PMC7486275 32494016

[39] X. Zhang , K. Yu , L. Ma , et al., “Endogenous Glutamate Determines Ferroptosis Sensitivity via ADCY10‐Dependent YAP Suppression in Lung Adenocarcinoma,” Theranostics 11 (2021): 5650–5674. 10.7150/thno.55482 PMC8058707 33897873

[40] J. Lin , L. Chen , D. Wu , J. Lin , B. Liu , and C. Guo , “Potential Diagnostic and Prognostic Values of CBX8 Expression in Liver Hepatocellular Carcinoma, Kidney Renal Clear Cell Carcinoma, and Ovarian Cancer: A Study Based on TCGA Data Mining,” Computational and Mathematical Methods in Medicine (2022): 1372879. 10.1155/2022/1372879 PMC9259361 35813444

[41] X. Song , W. Ning , J. Niu , G. Zhang , H. Liu , and L. Zhou , “CBX8 Acts as an Independent RNA‐Binding Protein to Regulate the Maturation of miR‐378a‐3p in Colon Cancer Cells,” Human Cell 34 (2021): 515–529. 10.1007/s13577-020-00477-w 33417156

[42] Z. Zhao , J. He , S. Qiu , et al., “Targeting PLK1‐CBX8‐GPX4 Axis Overcomes BRAF/EGFR Inhibitor Resistance in BRAFV600E Colorectal Cancer via Ferroptosis,” Nature Communications 16 (2025): 3605. 10.1038/s41467-025-58992-z PMC12003730 40240371

[43] Q. Meng , L. Li , and L. Wang , “High CBX8 Expression Leads to Poor Prognosis in Laryngeal Squamous Cell Carcinoma by Inducing EMT by Activating the Wnt/β‐Catenin Signaling Pathway,” Frontiers in Oncology 12 (2022): 881262. 10.3389/fonc.2022.881262 PMC9259798 35814427

[44] X. Cai , Y. Lv , J. Pan , et al., “CBX8 Promotes Epithelial‐Mesenchymal Transition, Migration, and Invasion of Lung Cancer Through Wnt/β‐catenin Signaling Pathway,” Current Protein & Peptide Science 25 (2024): 386–393. 10.2174/0113892037273375231204080906 38265409

