# Supplementary material for: WDR72 Promotes Neuroblastoma Stemness and Progression by Sequestering TRIM31‐Mediated Degradation of CBX8
Source: Adv Sci (Weinh). 2026 Jul 30:e76602. Online ahead of print. doi: 10.1002/advs.76602 (PMC13423489; doi:10.1002/advs.76602)
Supplement: Supplementary file 1 — Supporting File 1: advs76602‐sup‐0001‐SuppMat.docx. [file ADVS-9999-e76602-s001.docx]

**Supplementary table 1 The lists of genes for Figure 1A.**

Genes associated with CSC markers were obtained from GSE90789 and those correlated with high risk or poor prognosis of neuroblastoma patients were acquired from TARGET-NBL.

**Supplementary Figure S1-S11. Supplementary figure legends and supplementary figures.**

**Supplementary Figure S1 Correlation of four selected markers with prognosis of** **neuroblastoma patients**

A-D. Data from TARGET-NBL showed the correlation of KIRREL2 (A), NCAN (B), EPPK1 (C) and WDR72 (D) with prognosis of neuroblastoma patients.

**Supplementary Figure S2 WDR72 Promotes Proliferation, Invasion, and Metastasis While Inhibiting Apoptosis in NB cells**

A. RT-qPCR and Western blot detection of WDR72 knockdown efficiency. B-F. CCK8, colony formation, flow cytometry, and transwell assays to analyze the proliferation, apoptosis, and invasion/metastasis ability of cells after WDR72 knockdown. The data of A and C-F were analyzed via one-way ANOVA, while data of B were analyzed via Two-way repeated measures ANOVA. ^**^P<0.01.

**Supplementary Figure S3 WDR72 Overexpression Enhances Tumorigenicity in Subcutaneous Xenograft Models**

A. Tumor volume growth curve over time and representative images of excised tumors from the WDR72 overexpression and control groups. B. Comparison of tumor weight between the WDR72 overexpression and control groups. The data of A were analyzed via Two-way repeated measures ANOVA, while data of B were analyzed via Student's t-test. ^*^P<0.05, ^***^P<0.001.

**Supplementary Figure S4 m6A Regulatory Network Controlling WDR72 Expression**

A. Western blot analysis of METTL3, METTL14, and METTL16 protein levels in neuroblastoma adherent cells versus serially passaged spheroids. B. RT-qPCR quantification of FTO and ALKBH5 mRNA levels in spheroids versus adherent cells. C. Western blot validation of FTO and ALKBH5 protein expression. D. RT-qPCR analysis of IGF2BP1, IGF2BP2, and IGF2BP3 mRNA levels. E. IGF2BP1, IGF2BP2, and IGF2BP3 protein expression assessed by Western blot. F. Dual-luciferase reporter assay of WDR72 3’UTR activity under METTL14 or IGF2BP1 overexpression. The data of B and D were analyzed via one-way ANOVA, and data of F were analyzed via Student's t-test. ^*^P<0.05, ^**^P<0.01, ^***^P<0.001, ^****^P<0.0001, n.s. meant no significance.

**Supplementary Figure S5 CBX8 Is Highly Expressed in NB Metastatic Tissues and Exhibits Oncogenic Properties**

A. RT-qPCR detection of CBX8 expression in NB tissues with or without metastasis. B. Western blot analysis of CBX8 expression in randomly-selected pairs of non-metastatic and metastatic NB tissues. C. Tissue microarray evaluation of CBX8 expression in NB metastatic tissues, NB non-metastatic tissues, and adrenal gland tissues. D. Correlation analysis of protein levels between CBX8 and WDR72. E. Colony formation assay and Sphere formation assay were applied to analyze changes in the proliferation ability and the sphere-forming ability of adherent cells overexpressing CBX8 and cell spheroids with CBX8 knockdown. F-G. Colony formation assay and Sphere formation assay analyzed the rescuing impact of Flag or Flag-CBX8 on the proliferation ability and the sphere-forming ability of cell spheroids with CBX8 knockdown. The data of A, C and E were analyzed via Student's t-test, data of D were obtained via Pearson correlation analysis, while data of F and G were analyzed via two-way ANOVA. ^*^P<0.05, ^**^P<0.01, ^***^P<0.001, ^****^P<0.0001.

**Supplementary Figure S6 WDR72 Stabilizes CBX8 to Sustain NB Cell Stemness**

A. CHX inhibition of protein synthesis, Western blot analysis of CBX8 expression levels at different time points after overexpression of WDR72. B. Western blot analysis of the regulatory effect of WDR72 on CBX8 expression. C-D. Colony formation assay and sphere formation assay were conducted in WDR72-knockdown NB cells transfected with CBX8-overexpressing vector or empty control. E-F. Colony formation and sphere formation assays assessed the proliferation and sphere formation abilities of WDR72-overexpressed NB cells with or without CBX8 silence. The data of A were analyzed via Two-way repeated measures ANOVA, while data of C-F were analyzed via two-way ANOVA. ^**^P<0.01, ^***^P<0.001, ^****^P<0.0001.

**Supplementary Figure S7 TRIM31 Negatively Regulates CBX8 to Modulate Oncogenic Properties of Neuroblastoma Cells**

A. Co-IP experiment to analyze the interaction between CBX8 and WDR72 in sphere cells. B. Interference of TRIM31 and overexpression of CBX8 in adherent cells, Western blot analysis of the expression changes of stem cell markers ALDH1 and Nanog. C-D. Interference of TRIM31 and simultaneous overexpression of CBX8 in adherent cells, analysis of cell cloning and sphere-forming ability changes. The data of C-D were analyzed via two-way ANOVA. ^*^P<0.05, ^**^P<0.01.

**Supplementary Figure S8 WDR72 Inhibits TRIM31-Mediated Ubiquitination of CBX8**

A. CoIP analyzed the impact of TRIM31 overexpression on the ubiquitination of endogenous CBX8 protein in MG132-treated SK-N-SH and IMR-32 cells, while western blot estimated the changes in CBX8 level in SK-N-SH and IMR-32 cells after TRIM31 overexpression. B. Western blot analysis of the regulatory effect of WDR72 on TRIM31 in cell spheroids. C. CoIP analyzed the interaction of TRIM31 with CBX8 and WDR72 in adherent cells and cell spheroids. D. Co-IP analysis of the interaction between TRIM31 and CBX8 in adherent cells with overexpression of WDR72. E. Co-IP analysis of the interaction between TRIM31 and CBX8 in cell spheroids with interference of WDR72. F. IF analyzed the co-localization of WDR72 and TRIM31 in IMR-32 cells.

**Supplementary Figure S9 TRIM31 Is inversely correlated with CBX8 in NB Tissues**

A. Western blot detection of TRIM31 expression in randomly-selected pairs of non-metastatic and metastatic NB tissues. B. RT-qPCR detection of TRIM31 expression in all the collected NB tissues. C. IHC detection of TRIM31 expression in NB tissues (images of randomly-selected 3 pairs of non-metastatic and metastatic tissues were shown), and TRIM31 staining positivity in all tissues were calculated. D. IHC detection of CBX8 expression in NB tissues (images of above 3 pairs of non-metastatic and metastatic tissues were shown), and CBX8 staining positivity in all tissues were calculated. E. The correlation between CBX8 positivity and TRIM31 positivity in non-metastatic and metastatic NB tissues were analyzed via Pearson correlation analysis. The data of B-D were analyzed via Student's t-test. ^**^P<0.01, ^****^P<0.0001.

**Supplementary Figure S10 WDR72 Modulates K48 and K63 Ubiquitination of CBX8**

A-B. Immunofluorescence experiments to analyze the changes in the levels of CBX8-K48 and CBX8-K63 in adherent cells after overexpression of WDR72 or in cell spheroids after interference of WDR72.

**Supplementary Figure S11 Impact of TRIM31 and CBX8 Mutations on Clone and Sphere Formation**

A. Overexpression of TRIM31 or mutants (ΔR, C36A, or ΔC-C), Western blot analysis of the effects of different vectors on the expression of CBX8, ALDH1, and Nanog. B-C. Overexpression of TRIM31 or mutants (ΔR, C36A, or ΔC-C), analysis of cell proliferation and sphere-forming ability through colony and sphere formation assays. D-E. Colony and sphere formation assays to analyze the changes in cell proliferation and sphere-forming ability of adherent SK-N-SH cells under different conditions. The data of B-E were analyzed via one-way ANOVA. ^*^P<0.05, ^**^P<0.01.
