# Supplementary material for: WDR72 Promotes Neuroblastoma Stemness and Progression by Sequestering TRIM31‐Mediated Degradation of CBX8
Source: Adv Sci (Weinh). 2026 Jul 30:e76602. Online ahead of print. doi: 10.1002/advs.76602 (PMC13423489; doi:10.1002/advs.76602)
Supplement: Supplementary file 2 — Supporting File 2: advs76602‐sup‐0002‐FigureS1‐S11.zip [file ADVS-9999-e76602-s002.zip › Supplementary Figure S11.pdf]

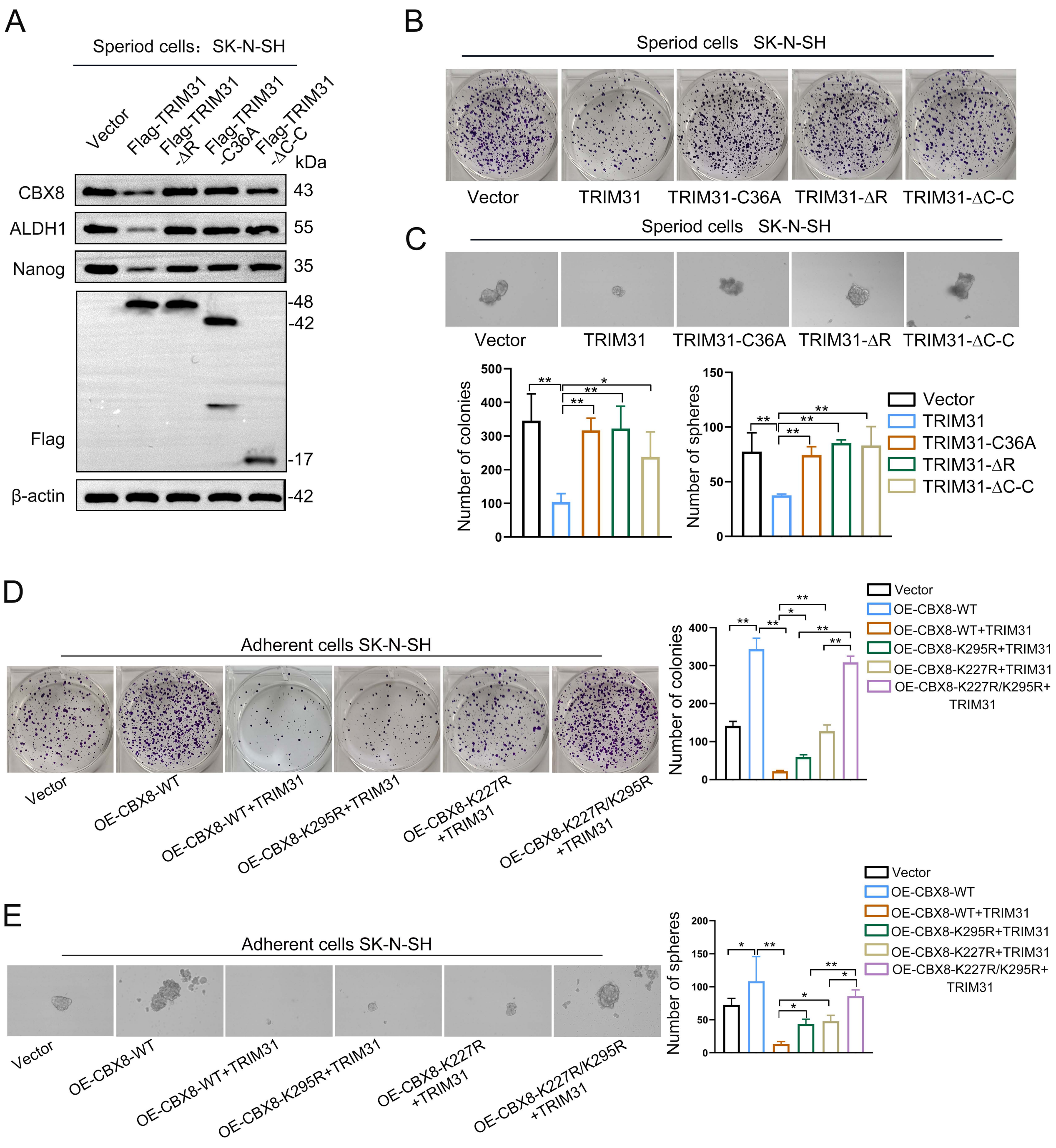

A
Speriod cells：SK-N-SH
Vector
Flag-TRIM31
Flag-TRIM31 -ΔR
Flag-TRIM31 -C36A
Flag-TRIM31 -ΔC-C
kDa
CBX8
43
ALDH1
55
Nanog
35
Flag
-48
-42
-17
β-actin
-42
B
Speriod cells SK-N-SH
Vector
TRIM31
TRIM31-C36A
TRIM31-ΔR
TRIM31-ΔC-C
C
Speriod cells SK-N-SH
Vector
TRIM31
TRIM31-C36A
TRIM31-ΔR
TRIM31-ΔC-C
Number of colonies
Number of spheres
Vector
TRIM31
TRIM31-C36A
TRIM31-ΔR
TRIM31-ΔC-C
D
Adherent cells SK-N-SH
Vector
OE-CBX8-WT
OE-CBX8-WT+TRIM31
OE-CBX8-K295R+TRIM31
OE-CBX8-K227R +TRIM31
OE-CBX8-K227R/K295R +TRIM31
Number of colonies
Vector
OE-CBX8-WT
OE-CBX8-WT+TRIM31
OE-CBX8-K295R+TRIM31
OE-CBX8-K227R+TRIM31
OE-CBX8-K227R/K295R+ TRIM31
E
Adherent cells SK-N-SH
Vector
OE-CBX8-WT
OE-CBX8-WT+TRIM31
OE-CBX8-K295R+TRIM31
OE-CBX8-K227R +TRIM31
OE-CBX8-K227R/K295R +TRIM31
Number of spheres
Vector
OE-CBX8-WT
OE-CBX8-WT+TRIM31
OE-CBX8-K295R+TRIM31
OE-CBX8-K227R+TRIM31
OE-CBX8-K227R/K295R+ TRIM31
