# Supplementary figures and images for: WDR72 Promotes Neuroblastoma Stemness and Progression by Sequestering TRIM31‐Mediated Degradation of CBX8
Source: Adv Sci (Weinh). 2026 Jul 30:e76602. Online ahead of print. doi: 10.1002/advs.76602 (PMC13423489; doi:10.1002/advs.76602)

A

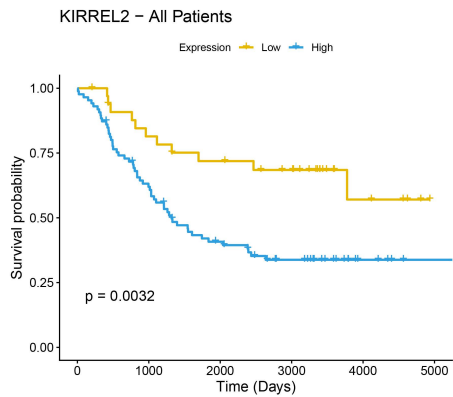

B

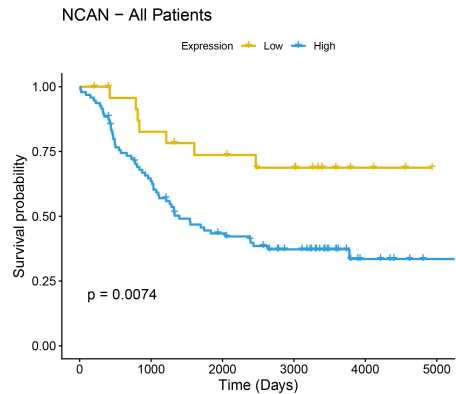

C

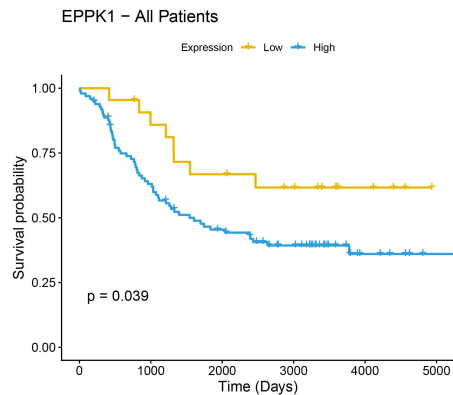

D

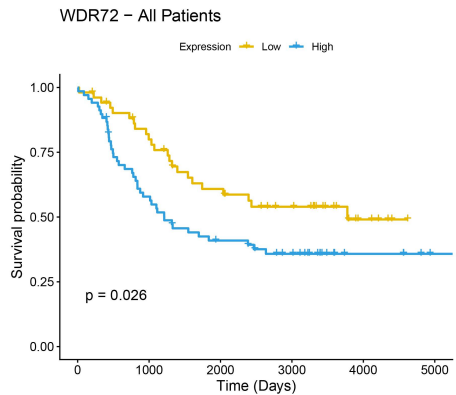

Supplement: Supplementary file 2 — Supporting File 2: advs76602‐sup‐0002‐FigureS1‐S11.zip [file ADVS-9999-e76602-s002.zip › Supplementary Figure S1.pdf]

A

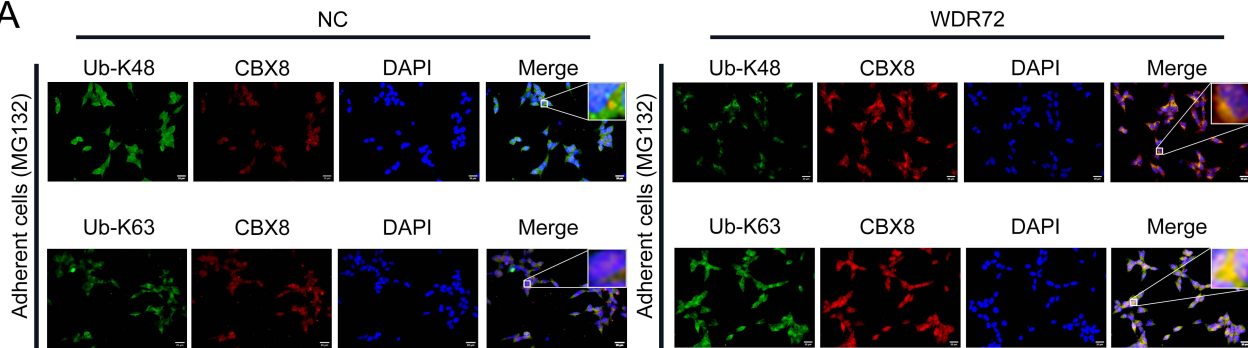

B

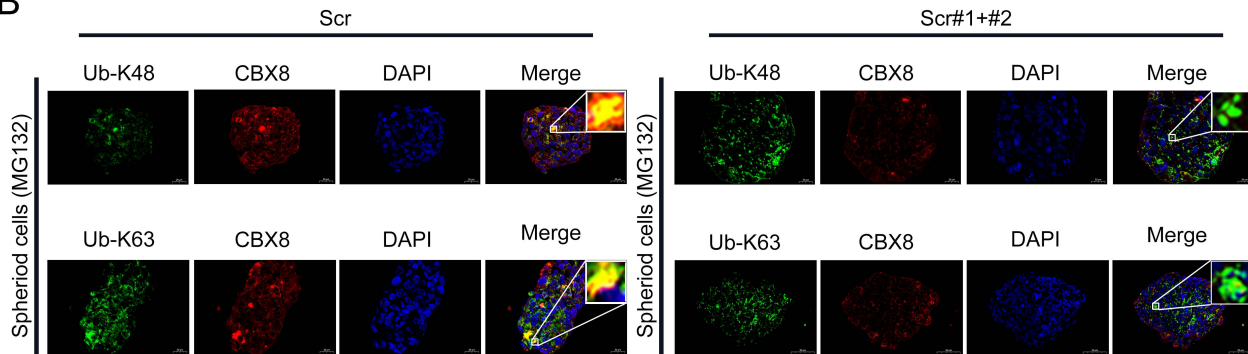

Supplement: Supplementary file 2 — Supporting File 2: advs76602‐sup‐0002‐FigureS1‐S11.zip [file ADVS-9999-e76602-s002.zip › Supplementary Figure S10.pdf]

**A**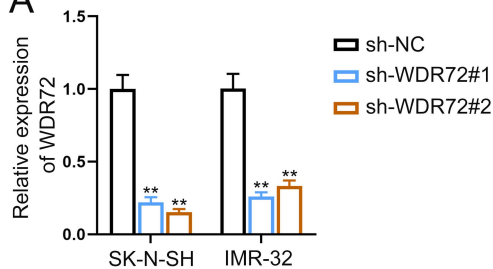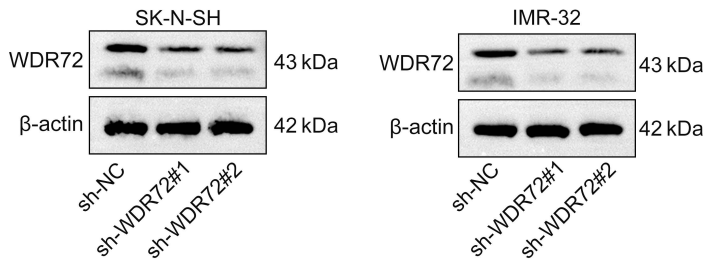**B**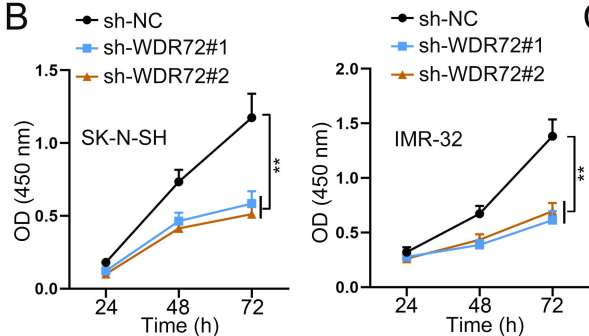**C**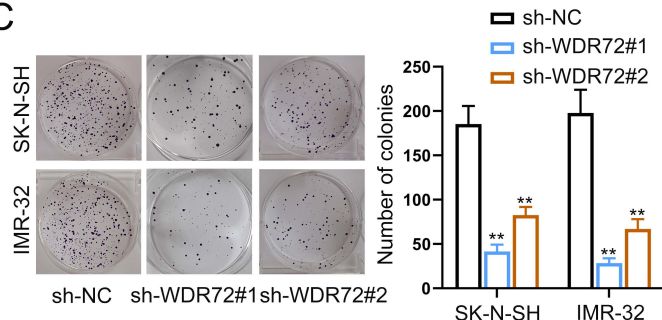**D**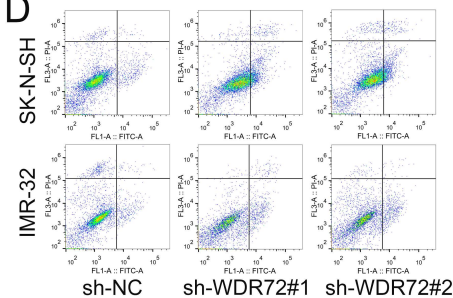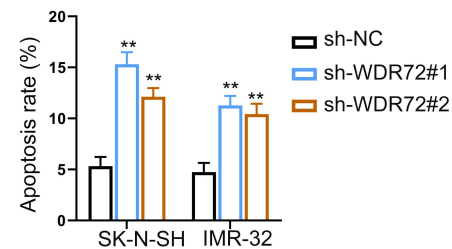**E**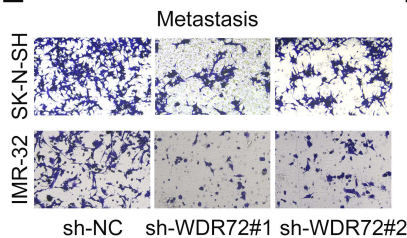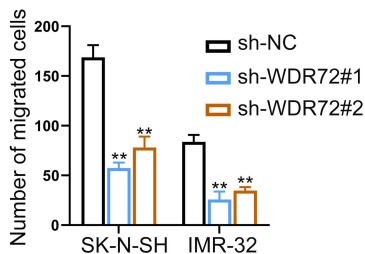**F**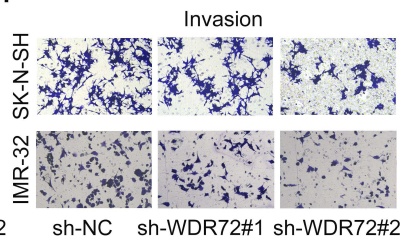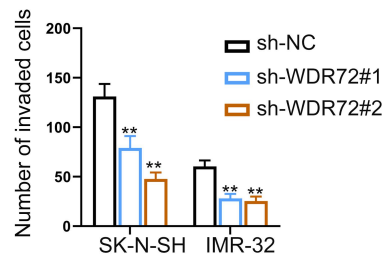

Supplement: Supplementary file 2 — Supporting File 2: advs76602‐sup‐0002‐FigureS1‐S11.zip [file ADVS-9999-e76602-s002.zip › Supplementary Figure S2.pdf]

**A**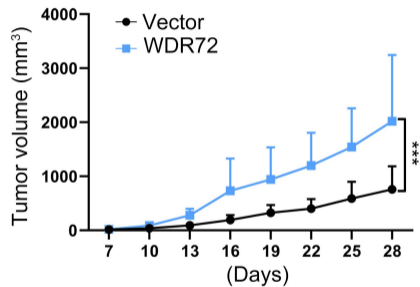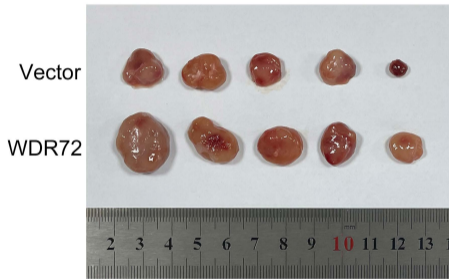**B**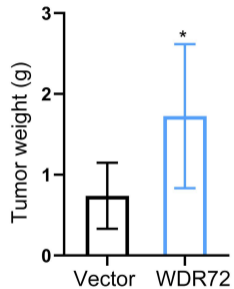

Supplement: Supplementary file 2 — Supporting File 2: advs76602‐sup‐0002‐FigureS1‐S11.zip [file ADVS-9999-e76602-s002.zip › Supplementary Figure S3.pdf]

**A**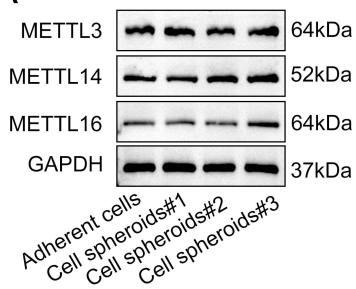**B**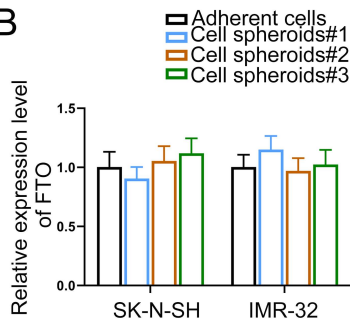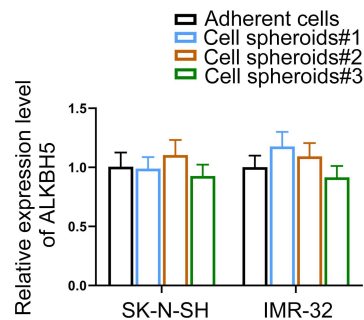**C**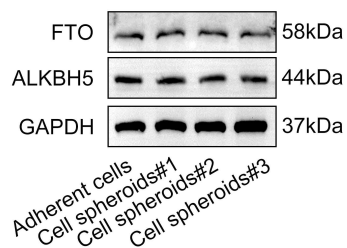**D**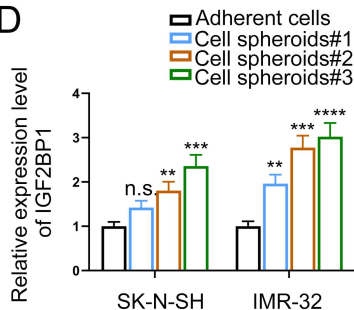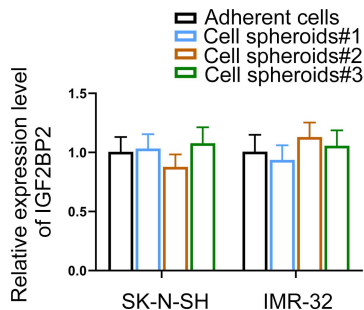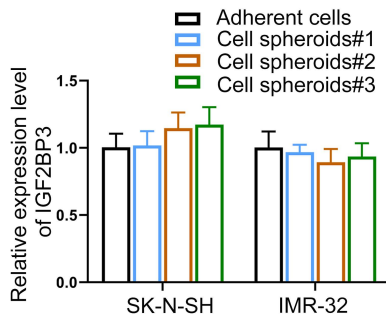**E**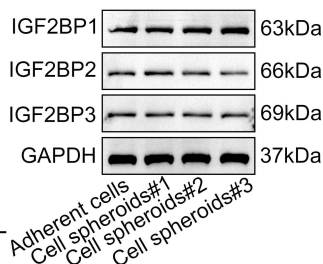**F**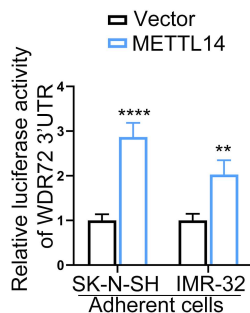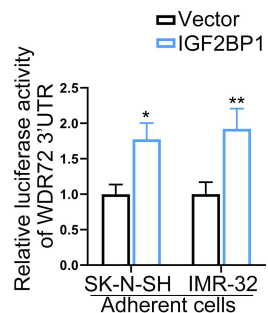

Supplement: Supplementary file 2 — Supporting File 2: advs76602‐sup‐0002‐FigureS1‐S11.zip [file ADVS-9999-e76602-s002.zip › Supplementary Figure S4.pdf]

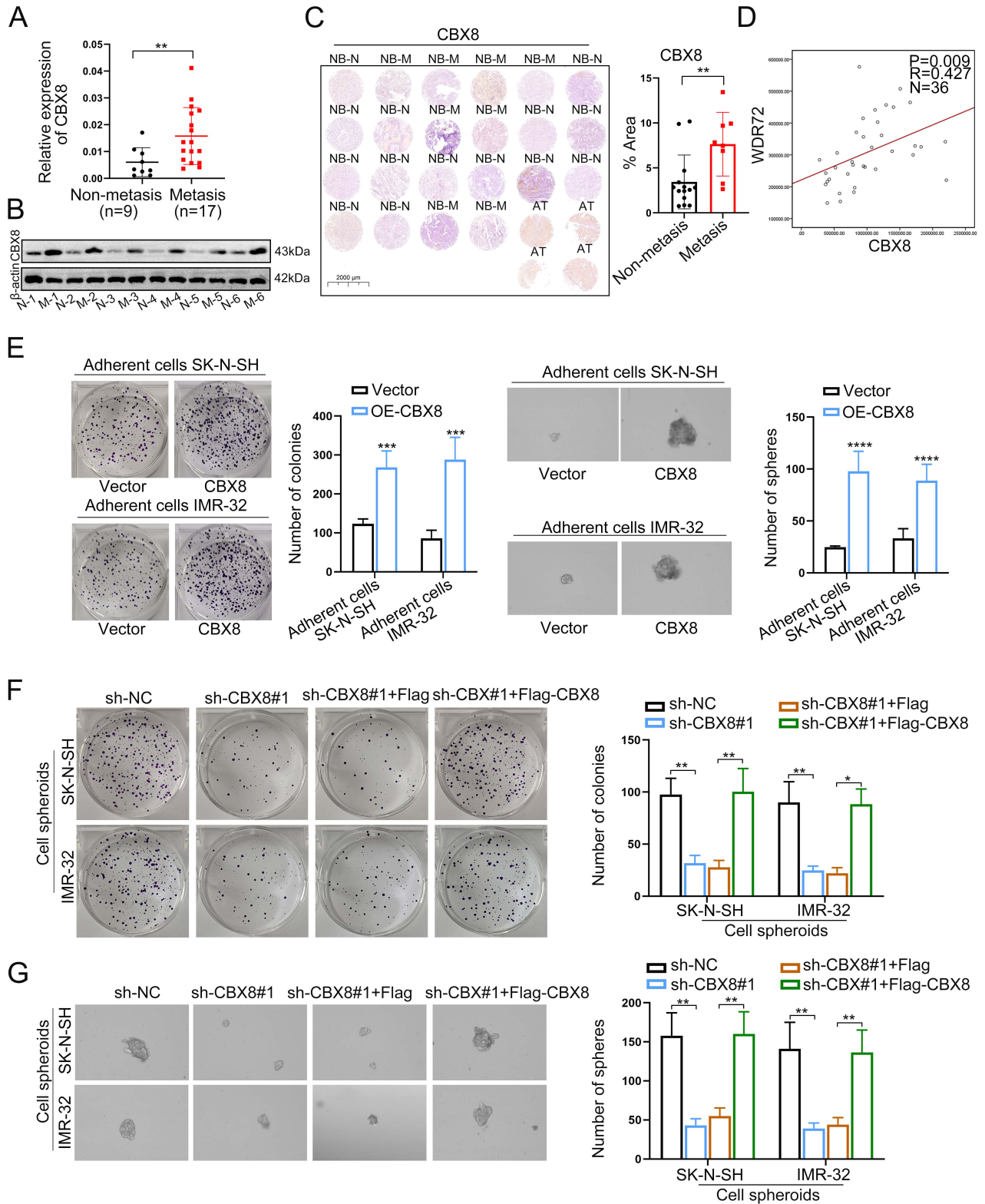

Supplement: Supplementary file 2 — Supporting File 2: advs76602‐sup‐0002‐FigureS1‐S11.zip [file ADVS-9999-e76602-s002.zip › Supplementary Figure S5.pdf]

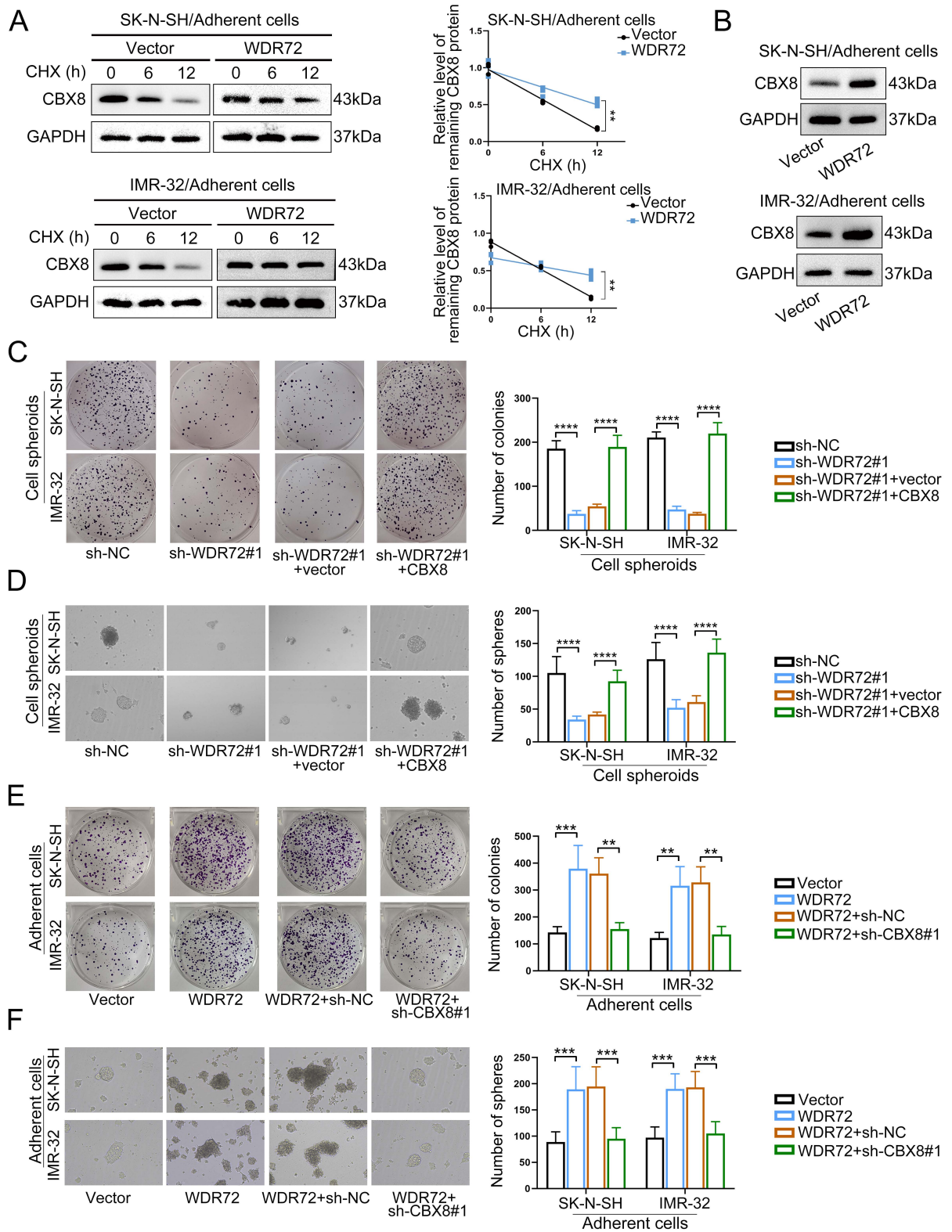

Supplement: Supplementary file 2 — Supporting File 2: advs76602‐sup‐0002‐FigureS1‐S11.zip [file ADVS-9999-e76602-s002.zip › Supplementary Figure S6.pdf]

A

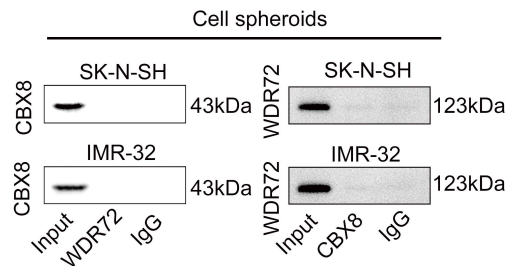

B

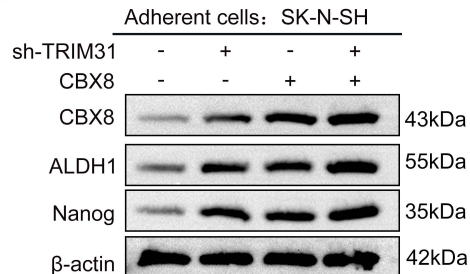

C

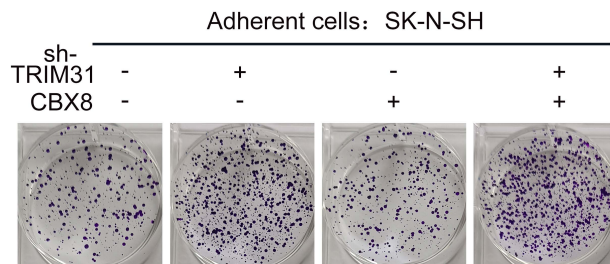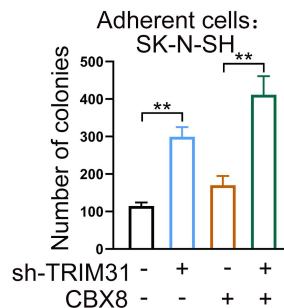

D

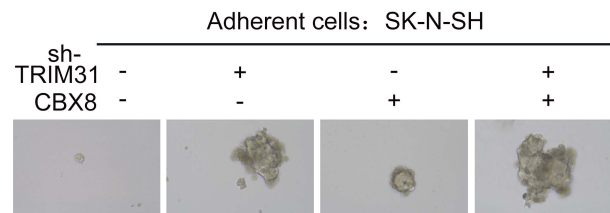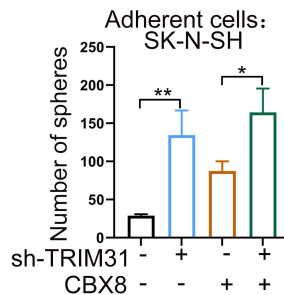

Supplement: Supplementary file 2 — Supporting File 2: advs76602‐sup‐0002‐FigureS1‐S11.zip [file ADVS-9999-e76602-s002.zip › Supplementary Figure S7.pdf]

A

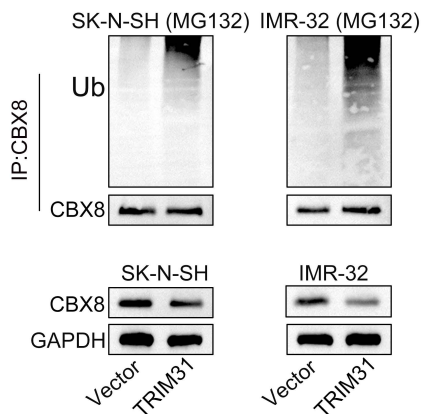

B

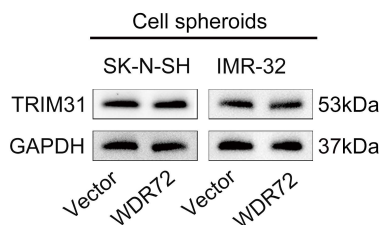

E

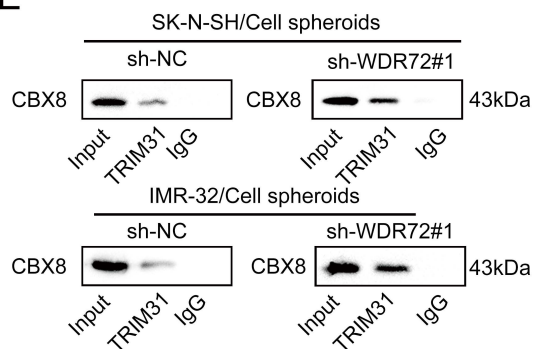

C

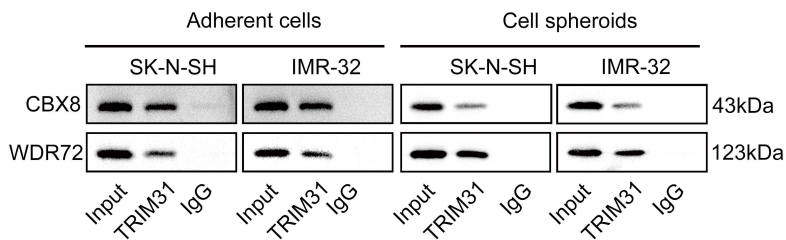

D

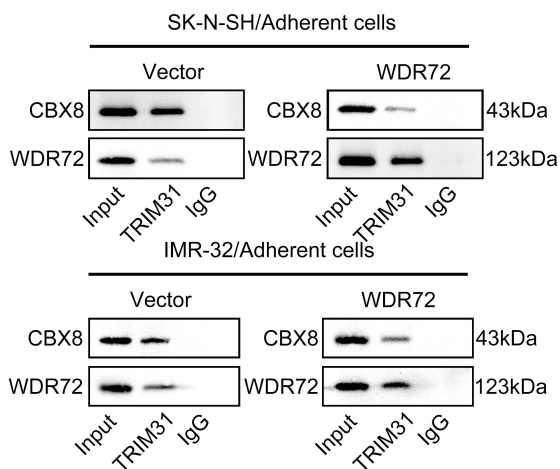

F

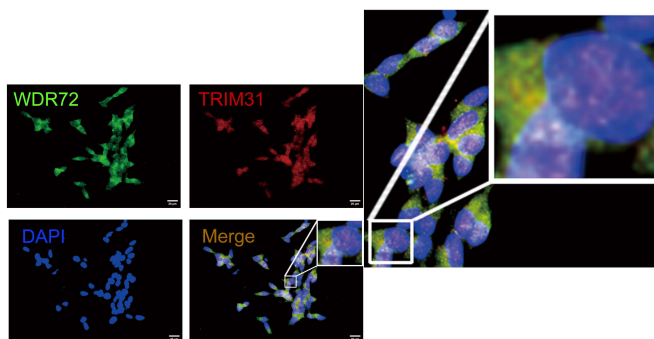

Supplement: Supplementary file 2 — Supporting File 2: advs76602‐sup‐0002‐FigureS1‐S11.zip [file ADVS-9999-e76602-s002.zip › Supplementary Figure S8.pdf]

A

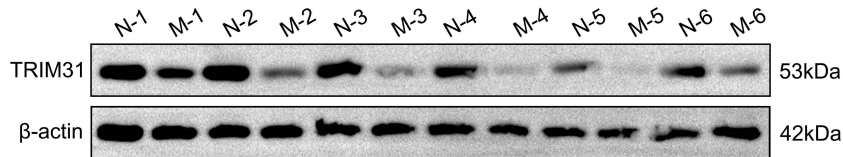

B

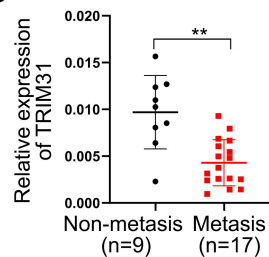

C

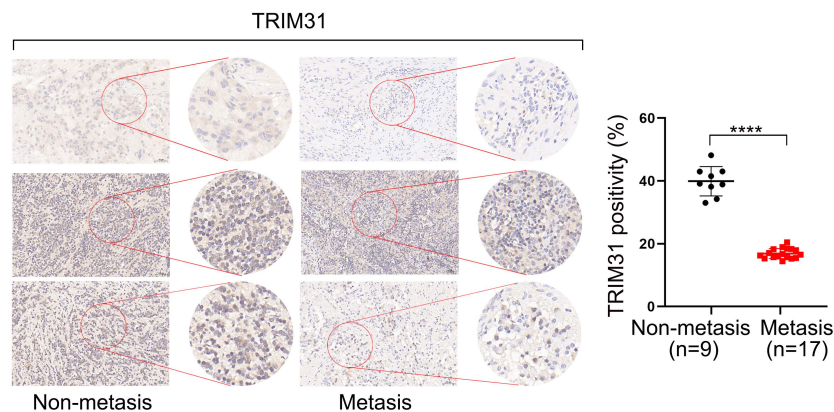

E

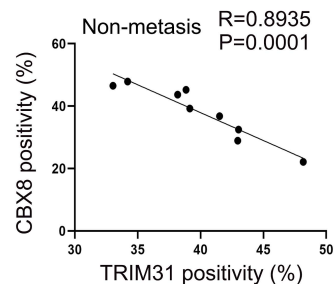

D

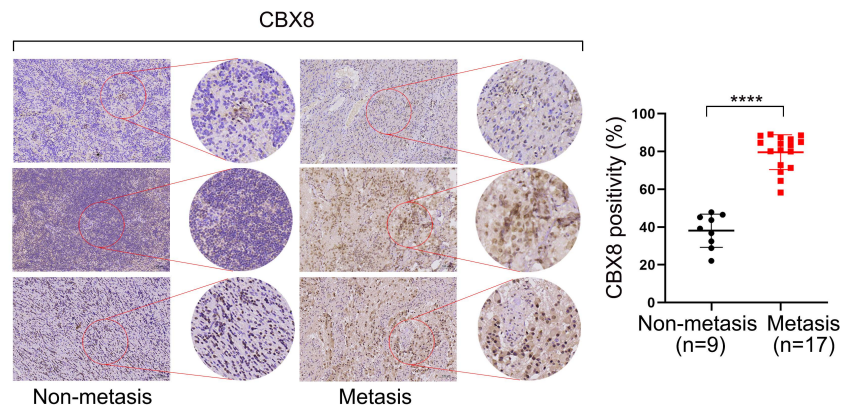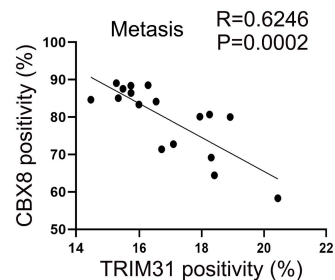

Supplement: Supplementary file 2 — Supporting File 2: advs76602‐sup‐0002‐FigureS1‐S11.zip [file ADVS-9999-e76602-s002.zip › Supplementary Figure S9.pdf]
